# Prohibitin-Mediated Lifespan and Mitochondrial Stress Implicate SGK-1, Insulin/IGF and mTORC2 in *C. elegans*

**DOI:** 10.1371/journal.pone.0107671

**Published:** 2014-09-29

**Authors:** Roxani Gatsi, Bettina Schulze, María Jesús Rodríguez-Palero, Blanca Hernando-Rodríguez, Ralf Baumeister, Marta Artal-Sanz

**Affiliations:** 1 CABD, Centro Andaluz de Biología del Desarrollo, CSIC-Universidad Pablo de Olavide-Junta de Andalucía, Sevilla, Spain; 2 Centre for Biological Signalling Studies (BIOSS), Albert-Ludwigs-University of Freiburg, Freiburg, Germany; 3 Laboratory for Bioinformatics and Molecular Genetics, Faculty of Biology, Albert-Ludwigs-University of Freiburg, Freiburg, Germany; 4 Center for Biochemistry and Molecular Cell Research, Faculty of Medicine, Albert-Ludwigs-University of Freiburg, Freiburg, Germany; National Center for Toxicological Research, US Food and Drug Administration, United States of America

## Abstract

Lifespan regulation by mitochondrial proteins has been well described, however, the mechanism of this regulation is not fully understood. Amongst the mitochondrial proteins profoundly affecting ageing are prohibitins (PHB-1 and PHB-2). Paradoxically, in *C. elegans* prohibitin depletion shortens the lifespan of wild type animals while dramatically extending that of metabolically compromised animals, such as *daf-2*-insulin-receptor mutants. Here we show that amongst the three kinases known to act downstream of *daf-2*, only loss of function of *sgk-1* recapitulates the ageing phenotype observed in *daf-2* mutants upon prohibitin depletion. Interestingly, signalling through SGK-1 receives input from an additional pathway, parallel to DAF-2, for the prohibitin-mediated lifespan phenotype. We investigated the effect of prohibitin depletion on the mitochondrial unfolded protein response (UPR^mt^). Remarkably, the lifespan extension upon prohibitin elimination, of both *daf-2* and *sgk-1* mutants, is accompanied by suppression of the UPR^mt^ induced by lack of prohibitin. On the contrary, gain of function of SGK-1 results in further shortening of lifespan and a further increase of the UPR^mt^ in prohibitin depleted animals. Moreover, SGK-1 interacts with RICT-1 for the regulation of the UPR^mt^ in a parallel pathway to DAF-2. Interestingly, prohibitin depletion in *rict-1* loss of function mutant animals also causes lifespan extension. Finally, we reveal an unprecedented role for mTORC2-SGK-1 in the regulation of mitochodrial homeostasis. Together, these results give further insight into the mechanism of lifespan regulation by mitochondrial function and reveal a cross-talk of mitochondria with two key pathways, Insulin/IGF and mTORC2, for the regulation of ageing and stress response.

## Introduction

Prohibitins are highly evolutionarily conserved proteins which act in a complex composed of two proteins, PHB-1 and PHB-2 [1]–[4]. Prohibitins interact with each other to form a ring-like heterodimeric superstructure situated on the inner membrane of the mitochondria [4], [5]. Several roles have been proposed for the prohibitin complex, suggesting it to act as a chaperone [2], to regulate mitochondrial protein degradation by modulating mitochondrial m-AAA protease activity [1], to stabilise the mitochondrial genome [6]–[8], to maintain mitochondrial integrity by stabilizing OPA-1 [9] and to act as a membrane scaffold to recruit membrane proteins [10]. However, the molecular mechanism of action of this complex remains a mystery. A more profound understanding of the biochemical function of prohibitins is of great importance as they are associated with the development of many human disorders such as cancer, inflammatory, cardiovascular and neurodegenerative diseases, and diabetes mellitus [11]. In this study, we use *Caenorhabditis elegans* as a model to study the function of prohibitins which amongst other phenotypes, have a remarkable effect on longevity as prohibitin depletion shortens the lifespan of wild type animals while, paradoxically, extending that of worms with metabolically compromised backgrounds [12]. To our knowledge, this differential effect on lifespan is inimitable and conserved through evolution [13], although other mitochondrial proteins are known to influence lifespan. The importance of mitochondrial function for the regulation of lifespan in *C. elegans* is evident by many paradigms of mitochondrial dysfunction promoting lifespan extension [14]–[17] whereas others result in lifespan shortening [18], [19]. Interestingly, it has been reported that a moderate reduction of mitochondrial protein function prolonged lifespan whereas a strong reduction resulted in lifespan shortening [20].

The induction of the mitochondrial unfolded protein response (UPR^mt^) initially emerged as of great importance for pro-longevity cues produced by long-lived mitochondrial mutants [21], [22]. Even though, in *C. elegans*, genes that when depleted induce the UPR^mt^ show a high correlation with extended lifespan [23], a recent work has shown that the UPR^mt^ is not required for lifespan extension [24]. Nevertheless, the UPR^mt^ has been implicated in extending the lifespan of worms, flies, and mice, suggesting a conserved role in cellular homeostasis [25]. Protein misfolding and aggregation induces the UPR^mt^ that leads to increased expression of mitochondrial chaperones for the recovery of mitochondrial homeostasis [26]. Moreover, the UPR^mt^ is induced by imbalance in the ratio of nuclear- and mitochondrial-DNA protein expression and this is involved in lifespan regulation [22]. Finally, the cellular surveillance-activated detoxification and defenses (cSADDs) [27] has been shown to regulate the ROS- triggered UPR^mt^ [28].

In *C. elegans*, prohibitin depletion strongly induces the UPR^mt^ [13], [29], [30]. Here, we investigated whether the UPR^mt^ is also implicated in lifespan regulation by prohibitins. To address this, we studied in more detail the genetic interaction of prohibitins with the insulin/IGF signalling (IIS) pathway in terms of lifespan regulation and induction of the UPR^mt^. Prohibitin elimination under reduced IIS, through mutations in the insulin receptor *daf-2*, prolongs lifespan by an astounding ∼150% and this increase is dependent on the *daf-16/*FOXO transcription factor [12]. The IIS pathway is well conserved among species; it is activated by the binding of insulin to its receptor (InR), encoded by *daf-2*. DAF-2 activates AGE-1, and the downstream kinases AKT-1, AKT-2 and SGK-1 [31]–[35]. Activation of AKT-1, AKT-2 and SGK-1, in turn phosphorylate and consequently inhibit the nuclear localization of DAF-16 [36]–[41]. Upon inhibition of the IIS cascade, DAF-16 is activated [40]–[42] and triggers the expression of various genes involved in the regulation of lifespan [43]. Our analysis of factors downstream of *daf-2* revealed that prohibitin depletion causes lifespan extension only in *sgk-1* mutant animals. Moreover, SGK-1 is acting in an additional pathway, parallel to DAF-2, for the regulation of lifespan upon prohibitin depletion. Remarkably, lifespan extension of both *sgk-1* and *daf-2* mutants was accompanied by a strong reduction of the UPR^mt^ induced by lack of prohibitins. In turn, we show that SGK-1 is acting together with RICT-1 for the induction of the prohibitin-mediated UPR^mt^ and that elimination of prohibitins extends the lifespan of *rict-1* loss of function mutants. *rict-1* encodes the *C. elegans* homologue of RICTOR protein, which is part of the mechanistic Target Of Rapamycin Complex 2 (mTORC2). Collectively, our data showed an inverse correlation of the induction of the UPR^mt^ and the extension of lifespan upon prohibitin depletion.

Our results not only contribute to a better understanding of ageing and the physiological function of prohibitins but also can provide valuable information for the development of therapeutic strategies to tackle prohibitin-associated diseases such as cancer, neurological, inflammatory, and metabolic diseases [11] as well as other age-related diseases.

## Results

### SGK-1 interacts with prohibitins to regulate lifespan

**Figure 1:**
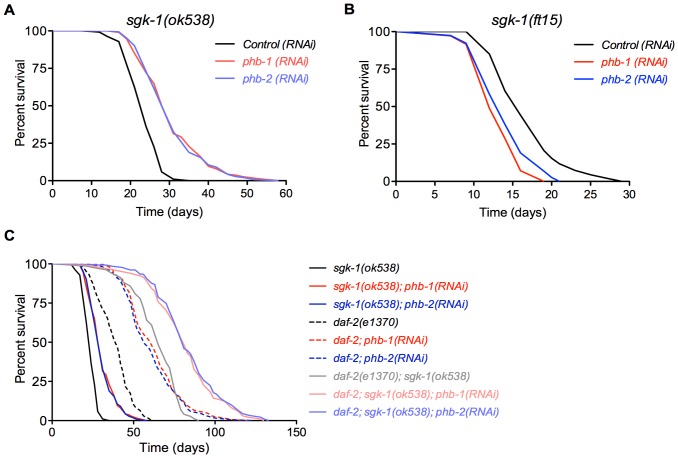
SGK-1 interacts with prohibitins to regulate lifespan. Lifespan curves are represented as the percentage of animals remaining alive against animal age (days). Combined lifespan data from independent experiments are shown in Table S1. A. Prohibitin depletion by RNAi against *phb-1* or *phb-2* at 20°C extends the lifespan of *sgk-1(ok538)* loss of function but not of *akt-1(ok525)*, *akt-2(ok393)* or *age-1(hx546)* (see Figure S1 and Table S1). B. Knockdown of *phb-1* or *phb-2* shortens the lifespan of *sgk-1(ft15)* gain of function. C. Prohibitin knockdown extends the lifespan of *daf-2(e1370)* and of the *daf-2(e1370); sgk-1(ok538)*, with the latest indicating additive effect.

Prohibitins have a peculiar effect on lifespan as prohibitin depletion causes lifespan shortening in a wild type background but conversely brings about a striking lifespan extension of ∼150% in a *daf-2* mutant background. This lifespan extension is *daf-16* dependent [12]. In an effort to understand how this differential regulation is achieved we investigated the interaction of prohibitins with other components of the insulin-like signalling pathway. Specifically we investigated the interaction with *age-1, akt-1, akt-2* and *sgk-1* encoding kinases. Intriguingly, we uncovered that *phb-1* and *phb-2* RNAi resulted in lifespan extension only in the *sgk-1(ok538)* mutant background (Figure 1A and Table S1), recapitulating the phenotype observed in *daf-2* mutants [12]. On the contrary, prohibitin depletion in *sgk-1* gain of function mutants, *sgk-1(ft15)*, caused shortening of lifespan (Figure 1B and Table S1). However, prohibitin depletion did not extend the lifespan of *akt-1(ok525)* (as already published [12]), *akt-2(ok393)* and *age-1(hx546)* loss of function mutant worms (Figures S1A, S1B, S1D and Table S1). Since double mutants of *akt-1* and *akt-2* arrest as dauers we could not address the possibility that they might be acting redundantly. Moreover, in the absence of SGK-1 it is possible that signalling is diverted through AKT-1/AKT-2, mediating the observed lifespan extension upon prohibitin depletion in the *sgk-1* null mutants. To address this, we investigated the effect of prohibitin elimination in *akt-1(mg144)* gain of function mutants; this allele has been shown to bypass the requirement of AGE-1 signalling in reproductive development [44]. If the lifespan extension upon prohibitin depletion in the absence of SGK-1 is due to up-regulation of signalling mediated through AKT-1/AKT-2, the *akt-1(mg144)* gain of function mutants would mimic this effect. However, we did not observe lifespan extension upon prohibitin depletion in *akt-1(mg144)* mutants (Figure S1C) indicating that lifespan extension upon prohibitin depletion in the *sgk-1(ok538)* animals is due to the loss of SGK-1 and not due to diversion of signalling through AKT-1/AKT-2. Although our results show that SGK-1 is the major kinase in the IIS pathway whose loss of function is required to mediate lifespan extension upon prohibitin depletion, we cannot exclude the contribution of AKT-1/-2.

### Lifespan of *sgk-1* mutants is affected by the DNA synthesis inhibitor, FUdR

Interestingly, in our hands *sgk-1(ok538)* mutants live longer than wild type animals on HT115 bacteria containing an empty RNAi vector. Over the years, there have been many contradictory results about whether SGK-1 has a promoting or inhibitory role for the regulation of lifespan [37], [45]–[48]. More recent data has shed light on this matter by showing that the effect of *sgk-1* mutation on lifespan depends not only on the food source but also on the temperature at which animals are raised [49]. We noticed that the studies reporting SGK-1 to have a promoting role for lifespan performed their assays with the addition of 5-fluoro-2-deoxyuridine (FUdR) [47]–[50]. In order to investigate if FUdR is responsible for this discrepancy we performed a lifespan assay of wild type and *sgk-1(ok538)* worms on HT115, with the addition or absence of FUdR. In accordance to our previous results, we found that *sgk-1(ok538)* animals live longer than wild type nematodes on HT115 in the absence of FUdR. Remarkably, this lifespan extension was suppressed by the addition of FUdR, however the mutant animals did not live shorter than the wild type control on FUdR (Figure S2 and Table S1). This might be attributed to other technical differences that could alter the responsiveness of *sgk-1* mutants, as these animals are known to be sensitive to differential environmental inputs [49]. Moreover, addition of FUdR did not affect the lifespan of wild type worms (Figure S2 and Table S1). Therefore, we conclude that the difference we observed with previous published work is partially due to the FUdR specifically affecting the *sgk-1(ok538)* mutants at 20°C, on HT115.

### SGK-1 is receiving input from an additional pathway, parallel to DAF-2, to interact with prohibitins for the regulation of lifespan

To get an insight into the interaction of prohibitins with SGK-1 and DAF-2 we tested the effect of *phb-1* and *phb-2* RNAi on the double loss of function mutant *daf-2(e1370); sgk-1(ok538)*. Remarkably, prohibitin depletion prolongs further the lifespan of the *daf-2(e1370); sgk-1(ok538)* double mutants reaching a striking 346% and 333% increase of mean lifespan upon *phb-1* and *phb-2* RNAi, respectively, compared to the wild type control (Figure 1C and Table S1). Our study also revealed that *sgk-1(ok538)* causes lifespan extension of the long-lived *daf-2(e1370)* animals (Figure 1C). This is in agreement with previously reported results showing lifespan extension of *daf-2(e1370)* animals subjected to *sgk-1* RNAi [37]. We enquired whether this extension is through the utilization of the IIS pathway, as *sgk-1* is also acting in other pathways [47], [49], [51]–[53]. The exceptional longevity of the *daf-2(e1370); sgk-1(ok538)* double mutant upon prohibitin depletion seems to be the additive effect of the lifespan extension individually conferred by prohibitin depletion to the *sgk-1* and the *daf-2* single mutants. The lifespan increase of the *daf-2; sgk-1* mutants on control RNAi is 236% while *phb-1* RNAi confers a 110% total increase to the individual single mutants (18% for *sgk-1* loss of function plus 92% for *daf-2* loss of function mutants). Hence the overall increase of lifespan upon prohibitin depletion, which is 346%, is the sum of the lifespan increase of the double *daf-2(e1370); sgk-1(ok538)* mutants and the increase individually conferred to the single mutants. These results suggest that SGK-1 is acting in a parallel pathway to DAF-2 to regulate lifespan extension upon prohibitin depletion. However, since *daf-2(e1370)* is a partial loss of function allele, we cannot exclude the contribution of lack of SGK-1 to the signalling mediated through DAF-2 for the extension of lifespan caused by lack of prohibitins (see bellow).

### Extension of lifespan in *daf-2* and *sgk-1* mutants upon prohibitin depletion inversely correlates with the induction of the UPR^mt^

**Figure 2:**
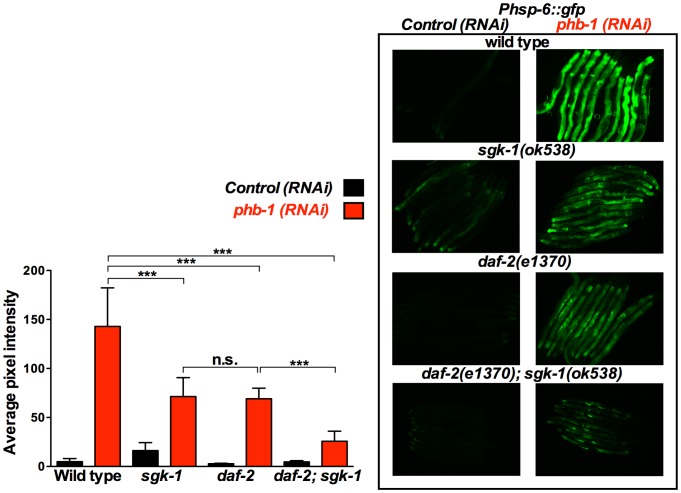
*sgk-1* and *daf-2* mutants suppress the prohibitin depletion-mediated induction of the UPR^mt^ reporter, *Phsp-6::gfp*. Fluorescent microscopy of *Phsp-6::gfp* animals subjected to control RNAi (empty vector pL4440) or *phb-1* RNAi (right panel) and graphical representation of the quantification of average pixel intensity under the corresponding conditions (left panel). Images were acquired under the same exposure, at the young adult stage. Prohibitin depletion at 20°C induced the UPR^mt^, as recorded by the mitochondrial chaperone reporter, *Phsp-6::gfp*. *daf-2(e1370)* and *sgk-1(ok538)* loss of function suppressed the prohibitin induced UPR^mt^. *daf-2(e1370); sgk-1(ok538)* double mutant caused an additive further suppression of the UPR^mt^, suggesting that *daf-2* and *sgk-1* are acting in parallel pathways to regulate the induction of the UPR^mt^ upon prohibitin depletion. *** P value <0.0001, n.s. not statistically significant difference. Error bars denote SD. P values were calculated by using the student's t-test.

**Figure 3:**
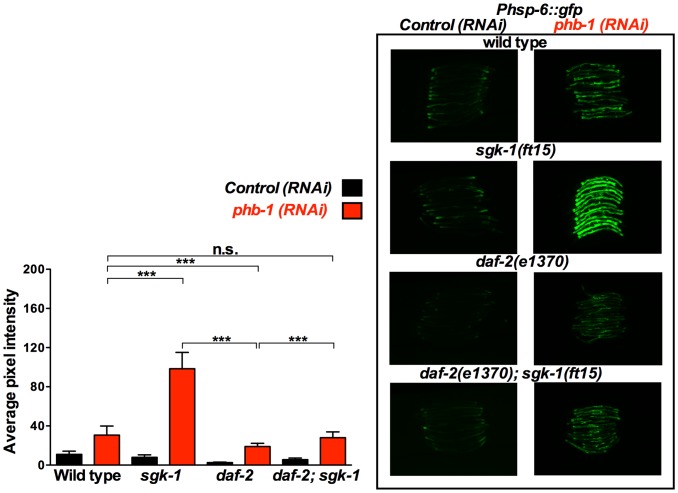
*sgk-1* gain of function enhances the prohibitin depletion-mediated induction of the UPR^mt^ in a *daf-2* dependent manner. Fluorescent microscopy of *Phsp-6::gfp* animals subjected to control RNAi (empty vector pL4440) or *phb-1* RNAi (right panel) and graphical representation of the quantification of average pixel intensity under the corresponding conditions (left panel). Worms were imaged at the young adult stage under the same exposure. *sgk-1(ft15)* gain of function at 20°C enhanced the prohibitin induced UPR^mt^, as recorded by the mitochondrial chaperone reporter, *Phsp-6::gfp*. *daf-2(e1370); sgk-1(ft15)* suppressed the effect of the *sgk-1* gain of function on the induction of the UPR^mt^ upon prohibitin depletion, suggesting that *daf-2* and *sgk-1* are acting in parallel pathways to regulate the induction of the UPR^mt^ upon prohibitin depletion. *** P value <0.0001, n.s. not statistically significant difference. Error bars denote SD. P values were calculated by using the student's t-test.

**Figure 4:**
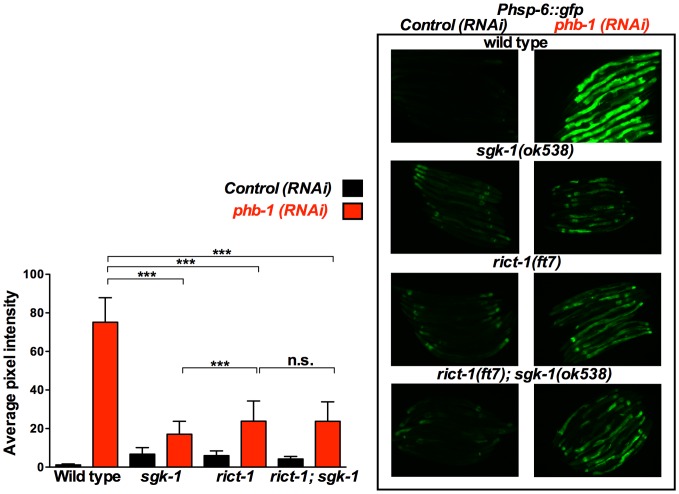
SGK-1 and RICT-1 act in the same pathway to regulate the prohibitin depletion-mediated induction of the UPR^mt^. Fluorescent microscopy of *Phsp-6::gfp* animals subjected to control RNAi (empty vector pL4440) or *phb-1* RNAi (right panel) and graphical representation of the quantification of average pixel intensity under the corresponding conditions (left panel). Worms were imaged at the young adult stage. *sgk-1(ok538)* and *rict-1(ft7)* loss of function at 20°C suppressed the prohibitin induced UPR^mt^ as recorded by the mitochondrial chaperone reporter, *Phsp-6::gfp*. *rict-1(ft7); sgk-1(ok538)* did not cause further suppression of the UPR^mt^, suggesting that *rict-1* and *sgk-1* are acting in the same pathway to regulate the induction of the UPR^mt^ upon prohibitin depletion. *** P value <0.0001, n.s. not statistically significant difference. *sgk-1(ok538)* and *rict-1(ft7)* mutants show increased *Phsp-6::gfp* expression when compared to wild type worms (*sgk-1(ok538*) vs. wild type: P<0.0001, *rict-1(ft7)* vs. wild type: P<0.0001, *rict-1(ft7); sgk-1(ok538)* versus wild type: P<0.0001). Error bars denote SD. P values were calculated by using the student's t-test.

**Figure 5:**
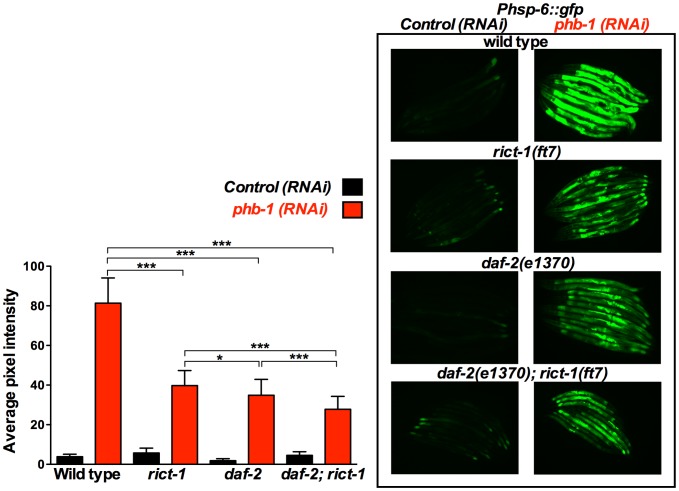
*rict-1* loss of function suppresses the prohibitin depletion-mediated induction of the UPR^mt^ in a parallel pathway to *daf-2*. Fluorescent microscopy of *Phsp-6::gfp* animals subjected to control RNAi (empty vector pL4440) or *phb-1* RNAi (right panel) and graphical representation of the quantification of average pixel intensity under the corresponding conditions (left panel). Worms were imaged at the young adult stage. *daf-2(e1370)* and *rict-1(ft7)* loss of function at 20°C suppressed the prohibitin induced UPR^mt^ as recorded by the mitochondrial chaperone reporter, *Phsp-6::gfp*. *daf-2(e1370); rict-1(ft7)* caused an additive further suppression of the UPR^mt^, suggesting that *daf-2* and *rict-1* are acting in parallel pathways to regulate the induction of the UPR^mt^ upon prohibitin depletion. *** P value <0.0001, * P value <0.01, n.s. not statistically significant difference. Error bars denote SD. P values were calculated by using the student's t-test.

Prohibitins have been suggested to act as mitochondrial chaperones involved in the stabilization of mitochondrial-encoded proteins [2] and in the regulation of the turnover of mitochondrial membrane proteins [1]. As such, prohibitin depletion strongly induces the UPR^mt^ (Figure 2, 3, 4, 5, S3 and [13], [29], [30]). Interestingly, the induction of the UPR^mt^ has been implicated in the generation of pro-longevity cues produced by long-lived mitochondrial mutants [21], [22]. However, recently it has been shown that the UPR^mt^ is not a predictor of longevity in *C. elegans* [24]. In order to understand the molecular mechanism(s) by which prohibitins regulate lifespan we questioned whether there is a link between the prohibitin-mediated regulation of lifespan and the UPR^mt^. Therefore, we investigated the UPR^mt^ effect of prohibitin depletion in *daf-2* and *sgk-1* mutants. We proceeded with the use of only the *phb-1* RNAi clone, since elimination of *phb-1* or *phb-2* by RNAi has a similar effect in lifespan (Figure 1, [12]) and on the induction of the UPR^mt^ (Figure S3), due to the fact that elimination of either prohibitin subunit results in the degradation of the respective assembly partner and the absence of the prohibitin complex [9], [54]–[58]. Intriguingly, prohibitin-induced overexpression of *Phsp-6::gfp*, a reporter commonly used for measuring the induction of the UPR^mt^, was suppressed in the long-lived *daf-2(e1370)* and *sgk-1(ok538)* loss of function mutants (Figure 2). Interestingly, the prohibitin mediated induction of the UPR^mt^ was further suppressed in the *daf-2(e1370); sgk-1(ok538)* double mutants (Figure 2), which shows the largest increase in lifespan, compared to the single mutants *daf-2(e1370)* and *sgk-1(ok538)* (Figure 1C).

On the contrary, *Phsp-6::gfp*, expression was enhanced in the *sgk-1(ft15)* gain of function mutants (Figure 3), which live shorter (Figure 1B), upon prohibitin depletion. This enhanced induction of the UPR^mt^ upon prohibitin depletion in the *sgk-1* gain of function mutants was suppressed by *daf-2(e1370)* (Figure 3), arguing against DAF-2 and SGK-1 acting exclusively in the same pathway, as one would expect the gain of function of SGK-1 to bypass the effect of DAF-2 loss of function. Collectively these data show that the extension of lifespan in *daf-2* and *sgk-1* mutants upon prohibitin depletion inversely correlates with the induction of the UPR^mt^.

### RICT-1 interacts with prohibitins to regulate lifespan and is signalling with SGK-1 for the regulation of the UPR^mt^

Our results described above indicate that SGK-1 is acting in an additional pathway, parallel to the IIS, for the regulation of lifespan and the UPR^mt^ upon prohibitin depletion. This directed us to investigate the interaction of prohibitins with RICT-1, the *C. elegans* homologue of RICTOR protein, which is part of the mTORC2 (mechanistic Target Of Rapamycin Complex 2). SGK-1 has been previously reported to act downstream of RICT-1 in the regulation of development, reproduction, body size, fat storage, stress resistance and lifespan [47], [49], [51], [52]. Comparable to *sgk-1(ok538)*, *rict-1(ft7)* mutant animals suppressed the high induction of *Phsp-6::gfp* expression upon prohibitin depletion (Figure 4). Moreover, this induction of the UPR^mt^ was further repressed in *daf-2; rict-1* animals (Figure 5), indicating that RICT-1 is acting parallel to DAF-2 to mediate the induction of the UPR^mt^. Most importantly, *sgk-1; rict-1* mutant animals showed a similar phenotype as to the *rict-1* single mutants in terms of the levels of suppression of the prohibitin induced UPR^mt^ (Figure 4). This implies that SGK-1 and RICT-1 act in the same pathway for the regulation of the UPR^mt^. Interestingly, *rict-1* loss of function mutant animals subjected to either *phb-1* or *phb-2* RNAi lived longer (Figure S4 and Table S1). Together these results suggest that RICT-1 and SGK-1 are interacting with prohibitins in a parallel pathway to DAF-2 for the regulation of the UPR^mt^ and possibly lifespan.

### mTORC2 and SGK-1 affect the UPR^mt^ in a food source dependent fashion

**Figure 6:**
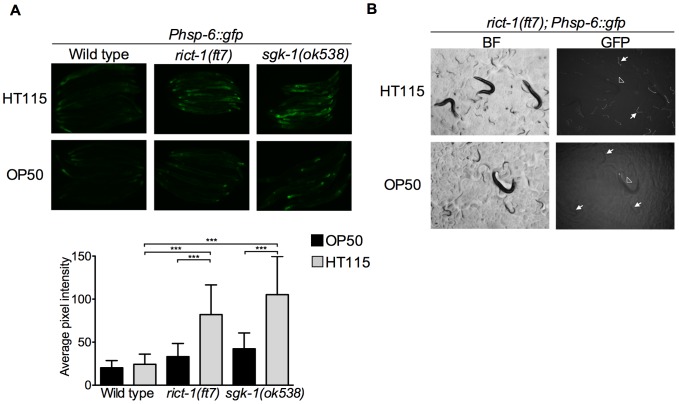
Induction of *Phsp-6::gfp* in *sgk-1* and *rict-1* mutants is food source dependent. Fluorescent microscopy of wild type; *Phsp-6::gfp*, *sgk-1(ok538); Phsp-6::gfp* and *rict-1(ft7); Phsp-6::gfp* animals grown on either HT115 or OP50 bacteria. A. Fluorescent images of wild type; *Phsp-6::gfp*, *sgk-1(ok538); Phsp-6::gfp* and *rict-1(ft7); Phsp-6::gfp* young adults (upper panel) and graphical representation of the quantification of average pixel intensity under the corresponding conditions (lower panel). *** P value <0.0001, n = 25–35. Error bars denote SD. P values were calculated by using the student's t-test. B. Fluorescent stereoscope images of *rict-1(ft7); Phsp-6::gfp* animals growing on either HT115 or OP50 bacteria. Bright field (BF) and fluorescent images are shown. Arrowheads point to P0 animals and arrows to F1 animals. The induced expression the *Phsp-6::gfp* reporter is evident in the P0 generation and becomes very strong in the F1 generation of *rict-1(ft7)* animals grown on HT115 bacteria.

Remarkably, we uncovered that *sgk-1(ok538)* and *rict-1(ft7)* mutant animals show increased expression of *Phsp-6::gfp* reporter as compared to wild type worms (Figure 4, *sgk-1(ok538)* and *rict-1(ft7)* vs. wild type: P<0.0001), showing for the first time an unprecedented role for mTORC2 and SGK-1 in modulating the mitochondrial stress response. Several food source dependent phenotypes, like fat storage, stress resistance and lifespan, have been reported for *sgk-1* and *rict-1* mutants [47], [49]. We therefore investigated whether the induction of the UPR^mt^ seen in both, *sgk-1* and *rict-1* mutants, is food source-dependent. Our results reveal that UPR^mt^ induction is evident in animals grown in HT115 bacteria (used for RNAi experiments) and very mildly in OP50 bacteria (the standard *C. elegans* food source) (Figure 6A). Both, *sgk-1* and *rict-1* mutants show a modest but statistically significant increase in *Phsp-6::gfp* expression in OP50 (Figure 6A; wild type vs. *rict-1(ft7)* P value  = 0.0002, wild type vs. *sgk-1(ok538)* P value <0.0001, unpaired *t*-test). Remarkably, the moderate induction of the UPR^mt^ observed in *rict-1(ft7)* and *sgk-1(ok538)* mutants raised in HT115 bacteria from the first larval stage becomes very strong in the F1 generation (Figure 6B and S5). Interestingly, the F1 generation animals have a very slow developmental rate (data not shown).

### mTORC2 and SGK-1 affect mitochondrial biogenesis/turnover

**Figure 7:**
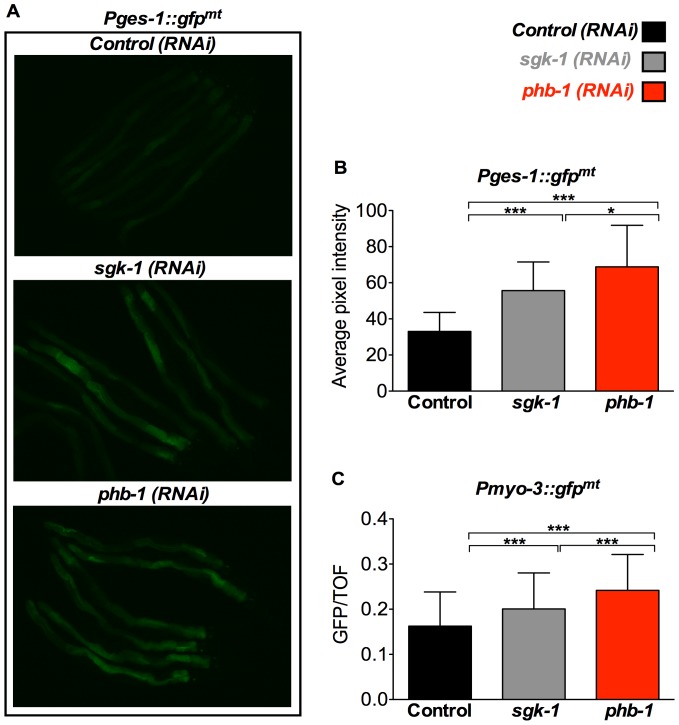
*sgk-1* and *phb-1* depletion by RNAi increases mitochondrial mass in the intestine and the body wall muscle. Analysis of mitochondrial content in *Pges-1::gfp^mt^* and *Pmyo-3::gfp^mt^* animals treated with empty vector pL4440 (control RNAi), *sgk-1* RNAi, or *phb-1* RNAi. A. Fluorescent microscopy of *Pges-1::gfp^mt^* animals. Worms were imaged at day 1 of adulthood. B. Graphical representation of the quantification of average pixel intensity under the corresponding conditions. Prohibitin and *sgk-1* depletion at 20°C increased intestinal mitochondrial mass as recorded by the intestinal mitochondrial reporter *Pges-1::gfp^mt^*. *** P value <0.0001, * P value <0.01. Error bars denote SD. P values were calculated by using the student's t-test. C. Graphical representation of the quantification of green fluorescence signal (GFP normalized to time of flight (TOF)) of *Pmyo-3::gfp^mt^* animals using the COPAS worm sorter. TOF and green fluorescence signal were recorded for each individual adult worm and was summarized by mean + SD. Worms were sorted at day 1 of adulthood. *** P value <0.0001, *n* = 100–200. Error bars denote SD. P values were calculated by using the student's t-test.

It has been reported that during the developmental time of *C. elegans* that coincides with mitochondrial biogenesis [59], there is a moderate increase of the UPR^mt^ [30]. Therefore, we questioned whether this small increase in the UPR^mt^ might reflect increased mitochondrial biogenesis. To assess this, we used the intestinal mitochondrial reporter *Pges-1::gfp^mt^*, which targets GFP into the mitochondria, and performed RNAi against *sgk-1*. Indeed, *sgk-1* RNAi treated worms show increased GFP in the intestine, reflecting increased mitochondrial content at day 1 of adulthood (Figure 7A and B). A similar increase in mitochondrial content upon depletion of *sgk-1*, was also observed using the body wall muscle mitochondrial reporter *Pmyo-3::gfp^mt^* (Figure 7C). Since RICT-1 and SGK-1 seem to act in the same pathway to regulate the UPR^mt^, we tested whether RNAi targeted against *rict-1* also increased mitochondrial content. In two out of four experiments *rict-1 (RNAi)* treated animals showed increased expression of the intestinal mitochondrial reporter *Pges-1::gfp^mt^* (Figure S6). This is, to our knowledge, the first report showing a role for mTORC2 and SGK-1 in the regulation of mitochondrial biogenesis/turnover. RNAi directed against *phb-1* also increased the expression of the mitochondrial reporters *Pges-1::gfp^mt^* (Figure 7A and B) and *Pmyo-3::gfp^mt^* (Figure 7C). This is in agreement with previous work showing increased mitochondrial content upon prohibitin depletion using Mitotracker staining [12]. Interestingly, although RNAi against both, *phb-1* and *sgk-1* result in increased mitochondrial mass (Figure 7), the induction of the UPR^mt^ upon *phb-1* RNAi was of much higher magnitude than that observed in the *sgk-1* mutants (Figure 2). This suggests that the rise in mitochondrial mass by loss of *sgk-1* reflects moderately stressed mitochondria while prohibitin depletion causes accumulation of highly malfunctioning/stressed mitochondria.

### mTORC2 and SGK-1 affect mitochondrial homeostasis

Mitochondria are the power source of cells as their main function is production of ATP. In an effort to investigate if *sgk-1* and *rict-1* mutant animals alter mitochondrial function we first quantified ATP levels. *sgk-1(ok538)* and *rict-1(ft7)* loss of function animals did not show a significant change in the total amount of ATP when compared to wild type control at the young adult stage (data not shown). Since alterations in ATP levels are not easily detectable at the young adult stage we also looked at day 10 of adulthood; in agreement, we did not observe any alteration in the ATP content of the mutant animals (Figure S7).

Membrane potential is crucial for the generation of ATP by ATP synthases. Therefore we quantified *in vivo* the mitochondrial membrane potential of day 1 adult animals by a fluorescence assay using the cationic, lipophilic carbocyanine dye, diS-C3 [60]. In accord with previously published work [61], we observed that the mitochondrial membrane potential is reduced in *daf-2(e1370)* mutant animals. However we did not observe any statistical differences in the *sgk-1(ok538)* and *rict-1(ft7)* loss of function animals compared to the wild type control (Figure S7).

**Figure 8:**
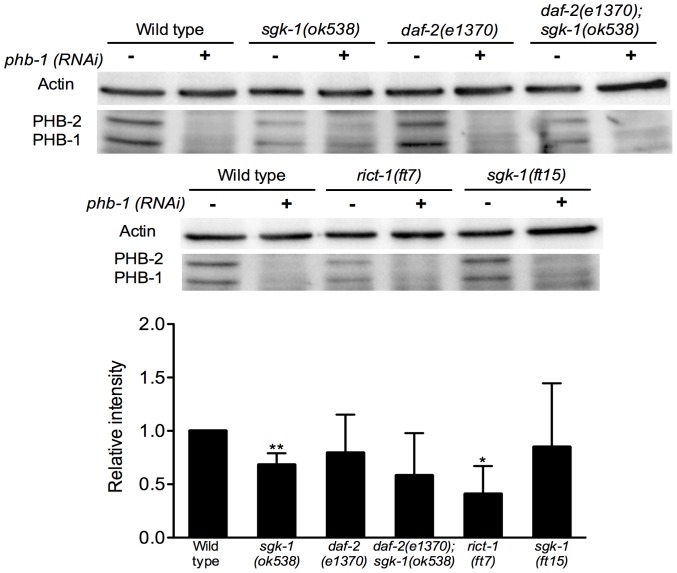
*sgk-1* and *rict-1* mutants have reduced levels of prohibitins. Western blot analysis showing actin, PHB-1 and PHB-2 protein levels of wild type, *sgk-1(ok538), daf-2(e1370), daf-2(e1370); sgk-1(ok538), rict-1(ft7) and sgk-1(ft15)* animals treated with *control RNAi* (*−*) or *phb-1* RNAi (+) (upper panel) and graphical representation of the quantification of PHB-1 average pixel intensity under the corresponding conditions normalized to the actin signal levels (lower panel). The data are represented as relative intensity normalized to the wild type control RNAi. PHB-1 and PHB-2 protein levels under *phb-1* RNAi were not detectable for the quantification. The antibody raised against PHB-1 recognizes also PHB-2 [12], [54]. The graph represents data from three independent experiments. Worms were grown at 20°C until young adult stage. *sgk-1(ok538)* and *rict-1(ft7)* mutants show a significant decrease in PHB-1 protein levels compared to the wild type control. *daf-2(e1370), daf-2(e1370); sgk-1(ok538)* and *sgk-1(ft15)* mutants do not have statistically significant different protein levels compared to wild type control. ** P value <0.05, *P value <0.1.

Although we did not observe any significant effect on mitochondrial function using the ATP and mitochondrial membrane potential assays, interestingly, Western Blot analysis revealed that *sgk-1(ok538)* and *rict-1(ft7)* mutants have reduced protein levels of PHB-1 (Figure 8). In contrast, *daf-2(e1370) and daf-2(e1370); sgk-1(ok538)* loss of function mutants did not show any alteration in the PHB-1 protein levels (Figure 8). Likewise, the gain of function of *sgk-1(ft15)* animals did not show an alteration in the protein content of PHB-1 (Figure 8).

Collectively, these results suggest that lack of SGK-1 and RICT-1 cause a reduction in the levels of prohibitins but this does not affect the ATP content and the mitochondrial membrane potential.

## Discussion

### SGK-1 is interacting with prohibitins to regulate longevity and stress response

Lifespan is differentially regulated by prohibitins as their depletion causes lifespan shortening in an otherwise wild type animals while, in a *daf-2* mutant background, results in lifespan extension [12]. The only kinase of the insulin pathway whose loss of function recapitulated this lifespan extension upon prohibitin depletion is SGK-1 (Figure 1). Although AGE-1 is directly receiving input from DAF-2, *age-1* loss of function did not cause lifespan increase by lack of prohibitins. The *age-1(hx546)* is a partial loss of function allele, therefore it is probable that the complete, or a stronger, loss of function allele is required for lifespan increase upon prohibitin depletion. *akt-1(ok525)* and *akt-2(ok393)* are null mutants, nonetheless, AKT-1 and AKT-2 have been reported to act redundantly for the regulation of dauer development [37], [44]. Therefore, we cannot exclude the possibility that in order to achieve lifespan extension upon prohibitin depletion the loss of function of both genes might be required. We could not test this as *akt-1; akt-2* mutants have a dauer constitutive phenotype. Nonetheless, the differential utilization of kinases in the IIS pathway for regulating distinct functions has been previously reported. SGK-1 has been shown to be of greater importance for the regulation of lifespan and oxidative stress resistance unlike AKT-1 and AKT-2 whose roles are more prominent for the regulation of dauer formation and the immunity response to pathogenic bacteria [37], [45]. Therefore, under mitochondrial stress such as upon prohibitin depletion, the organism might preferentially utilize SGK-1 to respond to these conditions. In agreement, recent data [49] has suggested that SGK-1 utilizes different transcription factors for the regulation of lifespan.

### SGK-1 receives input from RICT-1 to interact with prohibitins

SGK-1 is acting downstream of DAF-2 for the regulation of lifespan, development and stress resistance [37], [52]. However, in our study a series of observations suggested that SGK-1 is participating in signalling from an additional pathway to DAF-2 for the interaction with prohibitins. Primarily, the lifespan extension of the *daf-2; sgk-1* mutants resulting from prohibitin depletion was the additive effect of the longevity increase individually conferred by loss of prohibitins to the *sgk-1* and *daf-2* single mutants. Concurrently, the *daf-2; sgk-1* mutant animals showed an additive suppression of the UPR^mt^ triggered by prohibitin RNAi. Moreover, the strong enhancement of the prohibitin depletion-induced UPR^mt^ by the gain of function of *sgk-1* was suppressed in *daf-2* mutants. Arguing for a role of SGK-1 in parallel to the IIS, our study also revealed that *sgk-1 and daf-2* mutants behave differently. *sgk-1* loss of function induced the UPR^mt^, increased mitochondrial mass, caused a reduction in the levels of PHB-1 and did not affect ATP content and mitochondrial membrane potential (Figures 2, 4, S5, 6, 7, 8 and S7, respectively), in contrast to *daf-2* mutant animals which show a slight reduction or no effect of the expression of *Phsp-6::gfp* (Figures 2, 3 and 5), reduced intestinal mitochondrial content [12], no effect on the levels of PHB-1 (Figure 8), increase in ATP content [12] and reduction in mitochondrial membrane potential (Figure S7 and [12], [61]). Collectively, our results suggest that SGK-1 is signalling in an additional pathway parallel to DAF-2. Indeed, we uncovered that SGK-1 receives input from RICT-1 for the regulation of the prohibitin-induced UPR^mt^ (Figure 4). Moreover, we show that RICT-1 acts parallel to DAF-2 for the induction of the UPR^mt^ upon prohibitin depletion (Figure 5). In agreement, various sources have reported that SGK-1 functions downstream of RICT-1 for the regulation of fat metabolism, embryonic development, growth, stress resistance, lifespan, and dosage compensation mechanism [47], [49], [51], [52], [65]. Interestingly, prohibitin depletion confers longevity to *rict-1* mutant animals reminiscing the effect of the *sgk-1* mutants (Figures S4 and 1B, respectively). We propose that SGK-1 and RICT-1 are acting in the same pathway for the regulation of the UPR^mt^ and potentially lifespan upon prohibitin depletion.

### mTORC2 and SGK-1 affect mitochondrial homeostasis

Strikingly, lack of SGK-1 and RICT-1 trigger the induction of the reporter for the mitochondrial chaperone HSP-6 with the effect being more prominent on HT115 than on OP50 bacteria (Figure 6). Moreover, this induction of the UPR^mt^ is further enhanced in the progeny generated by the parents raised on HT115. Notably, the F1 generation also shows slower developmental rate, which is consistent with the slow growth rate observed by various mitochondrial mutants. Moreover, we observed that knockdown of *sgk-1* and *rict-1* by RNAi results in increased mitochondrial mass (Figures 7 and S6). This suggests that either SGK-1 and RICT-1 inhibit mitochondrial proliferation or lack of SGK-1 and RICT-1 trigger mitochondrial biogenesis. Alternatively, this increase in mitochondrial content could be attributed to a reduced elimination of mitochondria by mitophagy, although a role for SGK-1 in the regulation of mitophagy has, to our knowledge, not been reported. Interestingly, the mammalian orthologue of the stress-response transcription factor SKN-1, Nrf-2, promotes mitochondrial biogenesis and this requires its translocation to the nucleus [62]. Notably, the nuclear localization of SKN-1 in *C. elegans* is inhibited by SGK-1 [63], and more recent data has shown that RICT-1/mTORC2 negatively regulates longevity by inhibiting SKN-1/Nrf in the intestine through the SGK-1 kinase, which phosphorylates and inhibits SKN-1 [49]. This could account for the increased mitochondrial content observed in both, *rict-1* and *sgk-1* depleted animals.

Remarkably, addition of the DNA synthesis inhibitor, FUdR, suppressed the long lifespan of animals lacking SGK-1. Addition of FUdR could inhibit mitochondrial proliferation, as this process would require the replication of mtDNA [64]. Whether increase of mitochondrial stress and/or biogenesis is responsible for the lifespan extension of the *sgk-1* mutants deserves further investigation. Nonetheless, it is noteworthy that induction of the UPR^mt^ by lack of SGK-1 was more prominent when feeding animals with the bacterial food source HT115, reported to cause lifespan extension [47]. However, we cannot exclude the possibility that FUdR could indirectly affect the lifespan of the *sgk-1* mutants by altering the metabolism of the bacterial food source. The recent study by Mizunuma et al. [49] showed that FUdR does not shorten the lifespan extension conferred by *sgk-1(RNAi)* at 25°C, while we observe complete suppression of the extended longevity of *sgk-1(ok538)* mutants at 20°C. This discrepancy might be due to the differential effect of the mutation and the RNAi or plausibly due to an effect of the higher temperature. It is worth mentioning that the lifespan shortening phenotype of prohibitin depletion by RNAi is reverted at 25°C [12].

Surprisingly, *sgk-1* and *rict-1* loss of function mutants exhibited reduction in the levels of the mitochondrial protein PHB-1 even though mitochondrial content was increased in the corresponding mutants at day one of adulthood. As it has been shown in this paper and in agreement with previous work [12] prohibitin depletion increases mitochondrial number and induces the UPR^mt^. Therefore the moderate reduction of PHB-1 in the *sgk-1* and *rict-1* mutants could explain the increase of mitochondrial content and the mild induction of the UPR^mt^. Furthermore, *sgk-1* and *rict-1* mutants did not display any alteration in their ATP levels even though reduction of PHB-1 was observed. This observation is in agreement with an earlier report showing that depletion of prohibitins does not alter ATP content [12]. It is possible therefore that loss of SGK-1 and RICT-1 does affect mitochondrial function through regulation of prohibitins, however the increase of mitochondrial biogenesis/turnover restores normal levels of ATP. It would be of interest to investigate whether this down-regulation is due to a specific interaction of SGK-1 with PHB-1 and if a feedback mechanism exists.

### Extension of lifespan upon prohibitin depletion in *daf-2*, *sgk-1* and *rict-1* mutants: an inverse correlation with the induction of the UPR^mt^

Remarkably, the induction of the UPR^mt^ upon loss of prohibitins correlates with shortening of lifespan whereas its suppression in the *daf-2, sgk-1*, and *rict-1* mutant backgrounds promotes longevity. Induction of the UPR^mt^ has been reported to reflect the presence of stressed and/or dysfunctional mitochondria [30]. Prohibitins have been shown to have an imperative role in maintaining mitochondrial structure and function [10], [12]. The strong induction of the UPR^mt^ observed upon prohibitin depletion (Figures 2, 3, 4, 5, S3 and [13], [29], [30]) might be promoted by the accumulation of unfolded proteins, protein imbalance in the stoichiometry between PHB-1 and PHB-2 and possibly of other mitochondrial protein complexes, and finally by the generation of ROS. Moreover, accumulation of defective mitochondria, as a consequence of loss of prohibitins, would trigger the mitochondria retrograde response which would promote mitochondrial biogenesis [66]–[68]; hence the increased mitochondrial content observed upon prohibitin depletion (Figure 7, S6 and [12]). Here we show that strong induction of the UPR^mt^, as a result of prohibitin depletion in a wild type background, reflects severe mitochondrial dysfunction and correlates with reduction of lifespan. In agreement with this hypothesis, further induction of the prohibitin depletion-mediated UPR^mt^ in the *sgk-1* gain of function background results in additional reduction of lifespan. It has been shown that overexpression of SGK-1 inhibits massive autophagy [69]. Therefore, a plausible explanation is that defective mitochondria might accumulate in these mutants increasing mitochondrial stress and consequently the UPR^mt^. However, in a compromised metabolic background such as the *daf-2, sgk-1*, and *rict-1* loss of function mutants the severity of the prohibitin elimination effects are moderated, as observed by suppression of the UPR^mt^, while gradual decrease of the persistent UPR^mt^ correlates with continuing increase of lifespan in the corresponding mutant backgrounds. The less the prohibitin depletion-mediated UPR^mt^ is induced the longer the animals live. This would be in agreement with previous reports that showed that severe mitochondrial dysfunction can cause shortening of lifespan whereas mild defects can extend lifespan [20]. Although induction of the UPR^mt^ has been reported to be promoting lifespan extension [21], [22], [70], depletion of *phb-1/-2* are among the few cases in which induced UPR^mt^ correlates with shortening of lifespan [23]. Interestingly, a more recent publication shows no correlation between UPR^mt^ induction and lifespan. The authors report six additional RNAi clones, out of 19, that shortened lifespan despite inducing the UPR^mt^ [24]. Yet, induction of the UPR^mt^ reflects the presence of stressed/dysfunctional mitochondria [30]. Hence, there must be a threshold of the beneficial and the detrimental effects of mitochondrial stress measured by induction of the UPR^mt^. Strong mitochondrial defects in prohibitin depleted animals might trigger prolongevity cues however this is probably over-masked by the deleterious effects of mitochondrial dysfunction that the protective mechanisms of the cell cannot overcome, hereafter, leading to early death of the animals. These deleterious mitochondrial effects are diminished but not totally eliminated in the mutant backgrounds we have studied (as reflected by reduction but not total abolishment of the UPR^mt^). Under these conditions, the milder mitochondrial dysfunction upon prohibitin depletion could promote lifespan extension (Figure S8). Therefore, in the mutant backgrounds where prohibitin depletion causes lifespan extension there must be upregulation of cytoprotective mechanisms that would protect the organism from the deleterious effects of the severe mitochondrial dysfunction. The cytoprotective mechanisms in *C. elegans* involve up-regulation of autophagy, reduction of protein translation, generation of antioxidant and detoxification molecules, oxidative stress response, and induction of the cellular surveillance-activated detoxification and defense (cSADDs) mechanism [71]. Interestingly, *daf-2* mutant animals were recently reported to have reduced protein translation, including among others, HSP-6 [72]. SGK-1 has too been shown to promote protein synthesis in mammals [69]. Likewise, TOR which is part of mTORC1 and mTORC2 is promoting protein synthesis [73]. Therefore, it is possible that the suppression of the prohibitin-induced UPR^mt^ in the *daf-2*, *sgk-1* and *rict-1* mutant backgrounds is due to reduction of protein translation, which would ease the burden of incoming unfolded proteins into the mitochondria. This would be in agreement with recent reports suggesting that reduced cytoplasmic protein synthesis can be acting as a protective mechanism during mitochondrial dysfunction in human cancer cell lines, in yeast and in *C. elegans* [74]–[77]. Interestingly, reduced cytosolic protein synthesis suppressed aging-related mitochondrial degeneration in prohibitin mutants in yeast [8], [13]. Furthermore, our theory is further supported by the work of Schleit et al. [13] where it was shown that prohibitin depletion in *C. elegans* extends the lifespan of *rsks-1* mutants and of dietary restricted animals both of which show reduced cytoplasmic translation.

Another possible cytoprotective mechanism involved in lifespan extension upon prohibitin elimination in the *daf-2*, *sgk-1* and *rict-1* backgrounds might be mediated through induction of autophagy. Previous studies in *C. elegans* and other model organisms have reported that DAF-2, SGK-1 and mTOR inhibit autophagy [69], [73], [74], [78], [79]. In *C. elegans*, *sgk-1* depletion caused increase in autophagy in muscles, which was ascribed to increase in protein degradation [79]. Notably, in *C. elegans* autophagy and mitochondrial dynamics are required for removal and recovery of persistent mitochondrial DNA damage [80]. Increase in autophagy would also reduce protein content and amongst others eliminate dysfunctional mitochondria that can alleviate the deleterious effects of prohibitin depletion. Under these conditions, the milder mitochondrial dysfunction upon prohibitin depletion could trigger pro-longevity cues that can act beneficial for the organism and hence extend the lifespan of these animals. It is tempting therefore to speculate that increased autophagy and/or reduction of protein synthesis are protecting the organism from excessive mitochondrial damage caused by the knockdown of prohibitins. In effect, the reminiscing moderate mitochondrial dysfunction upon prohibitin depletion can lead to lifespan extension (Figure S8).

## Materials and Methods

### Strains

Standard procedures were followed for *C. elegans* strain maintenance. The following strains were used in this study: N2: wild-type Bristol isolate, BR927: *daf-2(e1370) III*, BR4774: *sgk-1(ok538) ×* (VC345 strain was obtained by CGC and outcrossed 8 times to N2), BR5749: *sgk-1(ft15) ×* (KQ1564 strain generated during an EMS mutagenesis screen [51] and then outcrossed 3 times to N2. After obtaining the strain directly from Kaveh Ashrafi/Kevin Jones we outcrossed it 6 more times to N2), BR3263: *age-1(hx546) II,* BR3037: *akt-1(ok525) V*, VC204: *akt-2(ok393) X*, GR1310: *akt-1(mg144) V*, BR5901: *rict-1(ft7) II* (KQ1366 strain generated during an EMS mutagenesis screen [51] and then outcrossed 3 times to N2. After obtaining the strain directly from Kaveh Ashrafi/Kevin Jones we outcrossed it 3 more times to N2), BR5185: *daf-2(e1370) III*; *sgk-1(ok538) X*, BR5194: *zcIs13[Phsp-6::gfp] V* (SJ4100, 7 times backcrossed against N2), BR6438: *zcIs13[Phsp-6::GFP] V*; *sgk-1(ft15) X*, BR6440: *zcIs13[hsp-6::GFP] V; sgk-1(ok538) X*, BR6296: *daf-2(e1370) III*; *(zcIs13[Phsp-6::gfp]) V*, BR6464: *rict-1(ft7) II; (zcIs13[Phsp-6::gfp]) V*, BR6465: *daf-2 (e1370) III*; (zcIs13[*Phsp-6::gfp*]) V; *sgk-1(ok538) X*, BR6463: *daf-2(e1370) III*; *(zcIs13[Phsp-6::gfp]) V; sgk-1(ft15) X*, BR6462: *rict-1(ft7) II; (zcIs13[Phsp-6::gfp]) V; sgk-1(ok538) X*, MRS65: *daf-2(e1370) III*; *rict-1(ft7) II; zcIs13[Phsp-6::gfp]) V*, SJ4143: *zcIs17[ges-1::GFP(mit)]* and SJ4103: *zcIs14[myo-3::GFP(mit)]*. Transgenic *Phsp-6::gfp* strains were generated by crossing BR5194 *(zcIs13[Phsp-6::gfp]) V* strain with the relevant mutants.

### RNAi assays

For RNAi experiments worms were placed on NGM plates seeded with HT115 (DE3) bacteria, containing 25 µg/ml carbenicillin, 5 µg/ml nystatin and either the pL4440 empty vector or the required target gene RNAi construct [12]. The RNAi bacterial cultures were incubated overnight in the presence of tetracycline and ampicillin. Next day, diluted cultures only containing ampicillin were grown at 37°C for 3 hours and 2 mM IPTG was added prior to seeding the plates and then induced at room temperature, overnight.

### Lifespan assays

All lifespan assays were conducted at 20°C and initiated with a synchronous embryo population on NGM plates containing the appropriate test bacterial strains. Synchronized eggs were obtained by adult hermaphrodites grown on OP50. Adult animals were transferred every day throughout their reproductive period and every 2–4 days thereafter. Animals were scored as dead when they stopped responding to touch, while ruptured animals or those that suffered internal hatching, extruded gonad, or desiccation due to crawling on the edge of the plates, were censored in the data analysis. We used the Prism software package (GraphPad Software) to plot survival curves by using the product-limit method of Kaplan and Meier. The log-rank (Mantel–Cox) test was used to evaluate differences between survivals and determine P values. For lifespans on FUdR, a synchronized embryo population was allowed to grow up to young adult stage in the absence of FUdR and then transferred on NGM plates containing 50 µM FUdR.

### Induction of the UPR^mt^

The induction of the UPR^mt^ was calculated by measuring the intensity of the *Phsp-6::gfp* reporter. Synchronized embryos were transferred on NGM plates seeded with HT115 (DE3) bacteria containing either the pL4440 empty vector or the *phb-1* RNAi construct. The animals were allowed to grow at 20°C until the young adult stage, when they were mounted on 2% agarose pads and imaged using an AxioCam MRm camera on a Zeiss ApoTome Microscope. Emission intensity was measured on greyscale images with a pixel depth of 16 bit. Average pixel intensity was calculated by sampling of 30-50 worms in each assay. Independent assays repeated three times. Image analysis was performed using the ImageJ software. Statistics were done using GraphPad Prism 4 software. The student's t-test was used to calculate P-values.

### Measurements of mitochondrial content

The mitochondrial intestinal content was calculated by measuring the intensity of the *Pges-1::gfp^mt^* reporter. Synchronized embryos were transferred on NGM plates seeded with HT115 (DE3) bacteria containing either the pL4440 empty vector or the appropriate RNAi construct [12], [37]. The animals were allowed to grow at 20°C until they were imaged (day 1 of adulthood). For the *Pges-1::gfp^mt^* reporter, animals were mounted on 2% agarose pads and imaged using an AxioCam MRm camera on a Zeiss ApoTome Microscope. Emission intensity was measured on greyscale images with a pixel depth of 16 bit. Average pixel intensity was calculated by sampling of approximately 30-40 worms in each assay. Independent assays repeated three times. Image analysis was performed using the ImageJ software.

The mitochondrial content in body wall muscle cells was calculated by measuring the intensity of the *Pmyo-3::gfp^mt^* reporter. Animals were treated as above until day 1 of adulthood. A COPAS Biosort system (Union Biometrica, Inc.) with Advances Acquisition Software Version 5.40.1.1 was utilized. Worms were washed from plates with sterile M9 and placed in the COPAS sample cup and analyzed. COPAS settings were as follows: gain extinction: 1; green: 1; threshold signal: 50; TOF minimum: 20; photomultiplier tube (PMT) setting control green: 400. Worms were gated based on TOF to select for adults. COPAS measured parameters [fluorescent channel 1 (Ch1) and time of fly (TOF)] were used to quantify mitochondrial content. GFP/TOF was calculated by sampling of 100–200 worms in each assay. Statistics were done using GraphPad Prism 4 software. The student's t-test was used to calculate P-values.

### ATP measurements

To determine ATP content, a semi-synchronous embryo population was raised on plates seeded with the appropriate RNAi bacterial clone at 20°C until they reached young or day 10 of adulthood. 50 worms were transferred to NGM plates without food and allowed to crawl for half an hour in order to remove excess of bacteria and then collected in 50 µl of S Basal buffer, fast-frozen in liquid nitrogen and stored at −80°C until further use. Frozen worms were immersed in boiling water for 15 min, cooled and centrifuged to pellet insoluble debris. The pellet was used to determinate total protein content. The supernatant was transferred to a fresh tube and diluted tenfold before ATP measurements. ATP content was determined by mixing 50 µl of the tenfold diluted sample with 50 µl of the luciferase reagent, included in the Roche ATP bioluminescent assay kit HSII (Roche Applied Science), and immediately the luminescence was measured using the POLARstar Omega luminometer (BGM Labtech). ATP levels were normalized to the total protein content of the corresponding sample. Independent assays repeated three times. Statistics were done using GraphPad Prism 4 software. The student's t-test was used to calculate P-values.

### Mitochondrial Membrane Potential measurements

Mitochondrial membrane potential was measured using the diS-C3 dye uptake method, adapted from Gaskova et al 2007 [60].

In brief, 100–150 day 1 adult worms were collected from plates with 5 ml of M9 buffer. The worms were washed twice with M9 and then resuspended in 5 ml of S-Basal buffer and incubated at 20°C for 30 min with gentle shaking. After washing with 5 ml of M9, the worms were resuspended in 2 ml of S-Basal buffer containing 4×10^−6^ M diS-C3 (freshly prepared), incubated for 80 min in a shaking incubator (120 rpm, 20°C). Following two more washes with 5 ml of M9, the worms were transferred on NGM plates without food, from where 15–30 worms were picked to be mounted on 2% agarose pads and imaged using an AxioCam MRm camera on a Zeiss ApoTome Microscope. Emission intensity was measured on greyscale images with a pixel depth of 16 bit. Image analysis was performed using the ImageJ software and the average pixel intensity was calculated in the terminal bulb of the pharynx. Statistics were done using GraphPad Prism 4 software. The student's t-test was used to calculate P-values.

### Protein content quantification

Total protein content was determined using the bicinchoninic acid (BCA) method previously described [81] with slight modifications. Briefly, the pellet from 50 worms was dried in a Speed Vac Concentrator (SPD12 1P SpeedVac, Thermo Scientific), 20 µl of 1 M NaOH was added to the dry pellet. Fat was degraded by heating at 70°C for 25 min and 180 µl of distilled water was added. After vortexing, the tubes were centrifuged at 14000 rpm for 5 min and 25 µl of the supernatant were transferred into a 96 well plate. Next, 200 µl of the BCA reagent prepared according manufacturer's instructions (Pierce BCA Protein Assay Kit, Thermo Scientific) and added to the sample. After incubation at 37°C for 30 min, the plate was cooled to room temperature and absorbance was measured using the POLARstar Omega luminometer (BGM Labtech) at 560 nm.

### Western Blots

Protein levels were quantified by immunoblot assay. A semi-synchronous embryo population was grown on plates seeded with the appropriate RNAi bacterial clone at 20°C until they reached young adult stage. 50 worms were transferred to NGM plates without food and allowed to crawl for half an hour in order to remove excess of bacteria and collected in 10 µl of M9 containing protease and phosphatase inhibitor cocktails (Roche Applied Science), fast-frozen in liquid nitrogen and stored at −80°C until further use. 10 µl of pre–heated sample buffer (0,5% Bromophenol Blue; 50% glycerol; 250 mM Tris pH 6,8; 10% SDS) was added to the sample, vortexed for 15 seconds, boiled 3 minutes at 95°C and loaded on a 12,5% SDS–PAGE gel and run in a Mini–PROTEAN Electrophoresis System (Bio–Rad). Following electrophoresis, proteins were transferred to a PVDF membrane (Immobilon, Millipore) using a wet Trans-Blot system (Bio-Rad). The immunoblots were visualized by chemiluminescent detection (SuperSignal, Thermo Scientific). Independent assays repeated three times. The chemiluminescent signals were quantified using the software ImageLab (Bio-Rad) and normalized to actin signal levels. The data are represented as relative values normalized to the wild type control. Statistics were done using GraphPad Prism 4 software. The student's t-test was used to calculate P-values.

Antibodies: A polyclonal antibody raised against the 25 carboxy-terminal amino acids of the murine PHB-1 protein has been described previously [3]. Anti-actin antibody was obtained from ICN (clone C4) and used at a dilution of 1∶10,000.

## Supporting Information

Figure S1 **Prohibitin depletion does not cause lifespan extension in the** ***akt-1, akt-2*** **and** ***age-1*** **mutant backgrounds.** Lifespan curves are represented as the percentage of animals remaining alive against animal age (days). Combined lifespan data from independent experiments are shown in Table S1. Prohibitin depletion by RNAi against *phb-1* or *phb-2*, at 20°C did not extend the lifespan of *akt-1(ok525)* loss of function (A); *akt-2(ok393)* loss of function (B); *akt-1(mg144)* gain of function (C); *age-1(hx546)* partial loss of function (D), suggesting that *akt-1, akt-2* and *age-1* are not involved in lifespan extension upon prohibitin depletion.
(PDF)

Figure S2 **Longevity conferred by loss of SGK-1 is dependent on FUdR, an inhibitor of DNA synthesis.** Lifespan curves are represented as the percentage of animals remaining alive against animal age (days). All animals were fed on HT115 bacteria with the addition of 50 µM FUdR where stated (+FUdR). *sgk-1(ok538)* mutants show lifespan increase in the absence of FUdR when compared to the wild type control, however, this longevity is suppressed by the addition of FUdR. The lifespan of wild type worms was not affected by the addition of FUdR.
(PDF)

Figure S3 ***phb-1*** **and** ***phb-2*** **RNAi induced the UPR^mt^.** Left panel: Fluorescent microscopy of *Phsp-6::gfp* and *Phsp-60::gfp* animals subjected to RNAi with either *phb-1* or *phb-2*. Worms were imaged at day 1 of adulthood. Depletion of either PHB-1 or PHB-2 induced similar levels of expression of the UPR^mt^ reporters *Phsp-6::gfp* and *Phsp-60::gfp*. Right panel: Quantification of average pixel intensity of *Phsp-6::gfp* and *Phsp-60::gfp* animals subjected to RNAi with either *phb-1* or *phb-2* (n = 20 per strain and condition).
(PDF)

Figure S4 **Prohibitin depletion extends the life span of** ***rict-1*** **loss of function animals.** Lifespan curves are represented as the percentage of animals remaining alive against animal age (days). Combined lifespan data from independent experiments are shown in Table S1. Prohibitin depletion by RNAi against *phb-1* or *phb-2*, at 20°C extended the lifespan of *rict-1(ft7)* loss of function mutants.
(PDF)

Figure S5 **Induction of** ***Phsp-6::gfp*** **in** ***sgk-1*** **mutants is more pronounced on HT115 in the F1 generation.** Fluorescent microscopy of wild type; *Phsp-6::gfp* and *sgk-1(ok538); Phsp-6::gfp* animals grown on either HT115 or OP50 bacteria. Fluorescent stereoscope images of wild type; *Phsp-6::gfp* and *sgk-1(ok538); Phsp-6::gfp* (P0) and their progeny (F1). Bright field (BF) and fluorescent images are shown. Arrowheads point to P0 animals and arrows to F1 animals (egg and larvae). The induced expression of the *Phsp-6::gfp* reporter is evident in the P0 generation and becomes very strong in the F1 generation of *sgk-1(ok538)* animals grown on HT115 bacteria.
(PDF)

Figure S6 ***rict-1*** **RNAi increases the mitochondrial mass in the intestine.** Fluorescent microscopy of *Pges-1::gfp^mt^* animals treated with empty vector pL4440 (control RNAi), or *rict-1* RNAi (right panel) and graphical representation of the quantification of average pixel intensity under the corresponding conditions (left panel). Worms were imaged at the day 1 of adulthood. *rict-1* depletion at 20°C increased intestinal mitochondrial mass as recorded by the intestinal mitochondrial reporter *Pges-1::gfp^mt^*. ** P value  = 0.0057 (n = 22 for control RNAi, n = 28 for *rict-1* RNAi).
(PDF)

Figure S7 ***sgk-1*** **,** ***rict-1*** **mutants do not effect ATP levels and the mitochondrial membrane potential.** Left panel. Graphical representation of the ATP content (mM ATP/µg protein) normalized relative to the wild type control. Animals grown on HT115 bacteria containing the empty vector pL4440 at 20°C until day 10 of adulthood when they were collected for quantification of ATP levels and protein content. The graph represents data from three independent experiments. *sgk-1(ok538)* and *rict-1(ft7)* mutants do not have statistically significant different ATP content compared to the wild type control. Right panel. Graphical representation of the average pixel intensity of diS-C3 dye uptake measured by fluorescent microscopy in day 1 adult animals grown on HT115 bacteria containing the empty vector pL4440 at 20°C. Data from one representative experiment are shown. *sgk-1(ok538)* and *rict-1(ft7)* mutants did not cause a statistical alteration in the mitochondrial membrane potential while *daf-2(e1370)* mutants show a significant decrease. *** P value <0.0001.
(PDF)

Figure S8 **Proposed model for the differential role of prohibitins on life span.** We propose that prohibitin depletion in a wild type background gives rise to severe mitochondrial dysfunction which over-induces mitochondrial stress response, resulting in early lethality for the organism. Conversely, in metabolically compromised background, like in *daf-2*, *sgk-1* and *rict-1* mutants, increased autophagy and/or reduction of protein synthesis is protecting the organism from excessive mitochondrial damage caused by the knockdown of prohibitins. This suppression of the mitochondrial damage/stress can be observed by suppression of the UPR^mt^. Under these conditions, the milder mitochondrial dysfunction upon prohibitin depletion could promote lifespan extension.
(PDF)

Table S1 **Summary of life span assays conducted for this study.** Unless otherwise stated, all ageing experiments were performed on plates seeded with HT115(DE3) *E. coli* bacteria, carrying appropriate RNAi plasmid constructs (SD: standard deviation of the mean). ¶Maximum lifespan shown is the median lifespan of the longest-lived 10% of the animals assayed. †The number of confirmed death events, divided by the total number of animals included in lifespan assays is shown. Total equals the number of animals that died plus the number of animals that were censored (see Methods). The number of independent lifespan assays for each strain is shown in parentheses. *Compared to wild type animals subjected to control RNAi. ‡Compared to the corresponding mutant subjected to control RNAi. P values were calculated using the Log-rank (Mantel-Cox) Test. ∞Compared to wild type animals on HT115. n.s: not significant statistical difference.
(PDF)
