# Supplementary figures and images for: Prohibitin-Mediated Lifespan and Mitochondrial Stress Implicate SGK-1, Insulin/IGF and mTORC2 in C. elegans
Source: PLoS One. 2014 Sep 29;9(9):e107671. doi: 10.1371/journal.pone.0107671 (PMC4180437; doi:10.1371/journal.pone.0107671)

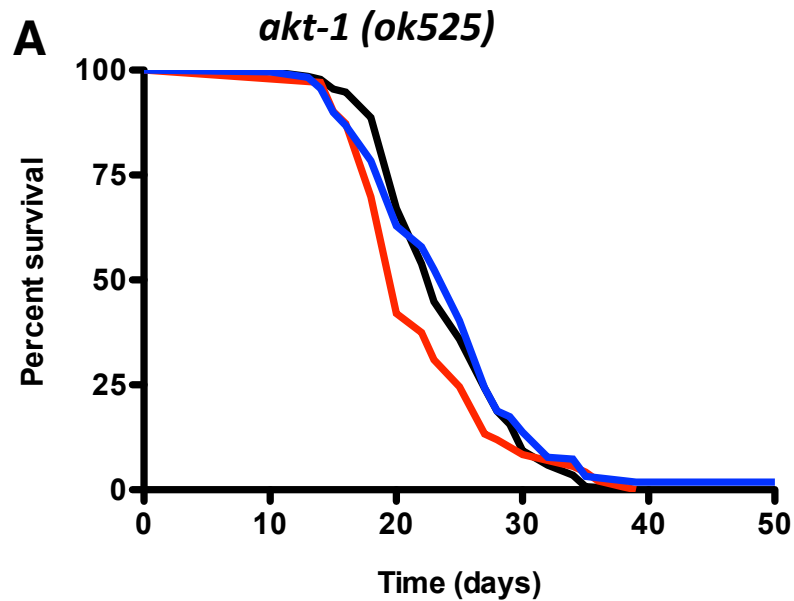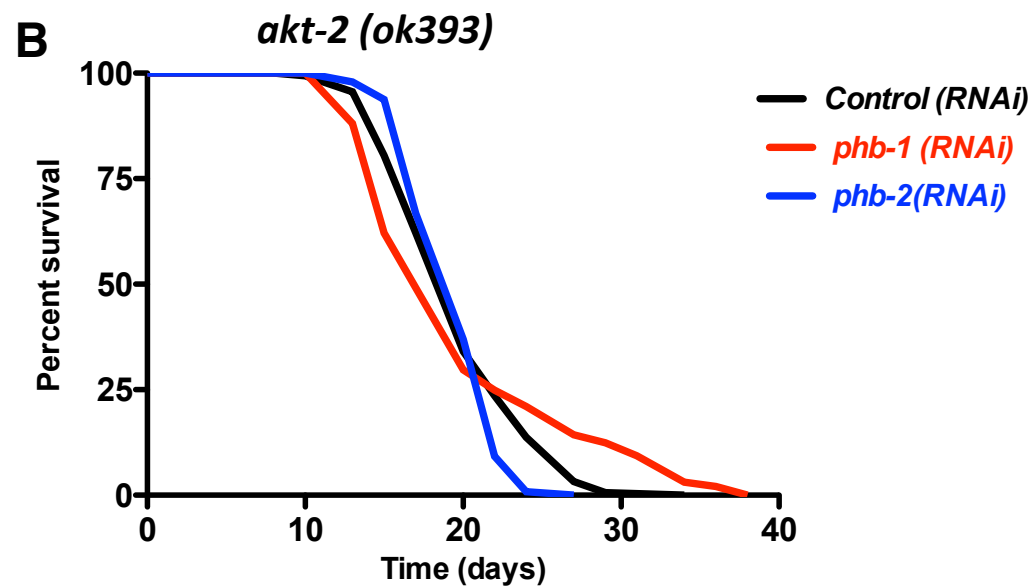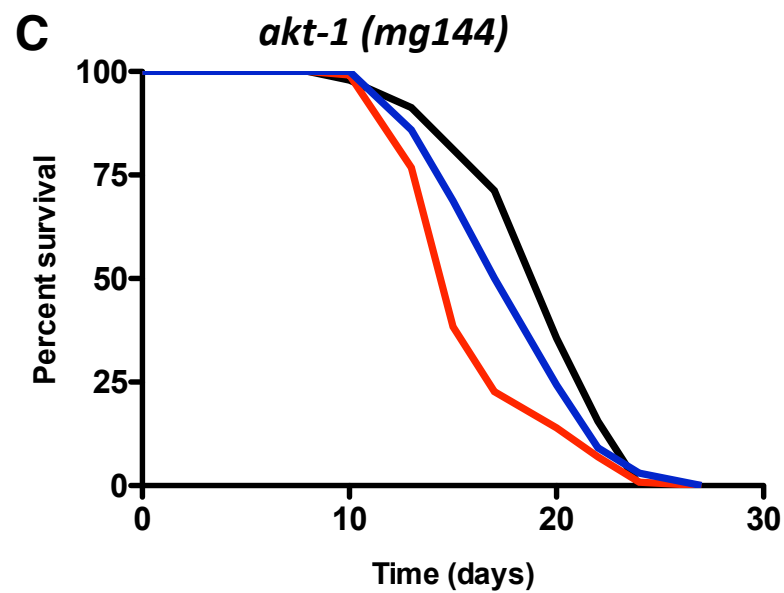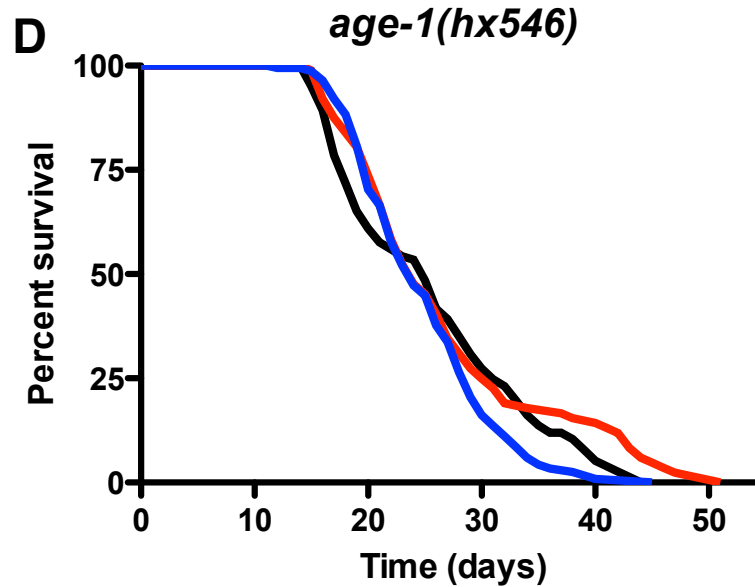

Supplement: Figure S1 — Prohibitin depletion does not cause lifespan extension in the akt-1, akt-2 and age-1 mutant backgrounds. Lifespan curves are represented as the percentage of animals remaining alive against animal age (days). Combined lifespan data from independent experiments are shown in Table S1. Prohibitin depletion by RNAi against phb-1 or phb-2, at 20°C did not extend the lifespan of akt-1(ok525) loss of function (A); akt-2(ok393) loss of function (B); akt-1(mg144) gain of function (C); age-1(hx546) partial loss of function (D), suggesting that akt-1, akt-2 and age-1 are not involved in lifespan extension upon prohibitin depletion. (PDF) [file pone.0107671.s001.pdf]

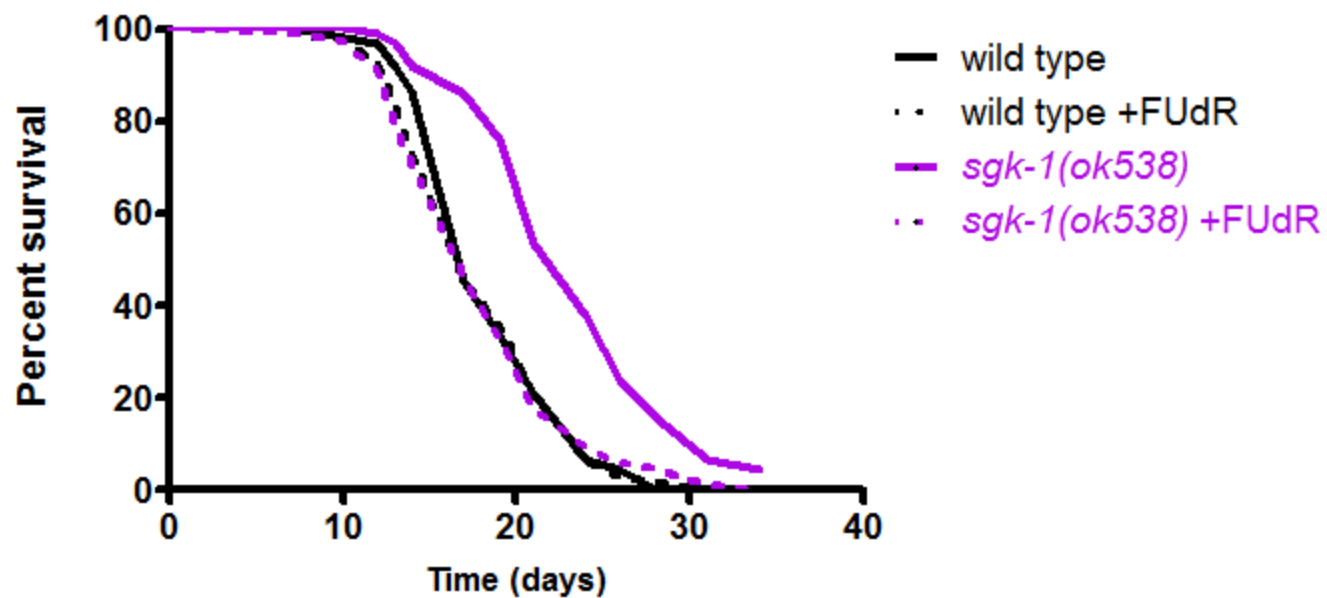

Supplement: Figure S2 — Longevity conferred by loss of SGK-1 is dependent on FUdR, an inhibitor of DNA synthesis. Lifespan curves are represented as the percentage of animals remaining alive against animal age (days). All animals were fed on HT115 bacteria with the addition of 50 µM FUdR where stated (+FUdR). sgk-1(ok538) mutants show lifespan increase in the absence of FUdR when compared to the wild type control, however, this longevity is suppressed by the addition of FUdR. The lifespan of wild type worms was not affected by the addition of FUdR. (PDF) [file pone.0107671.s002.pdf]

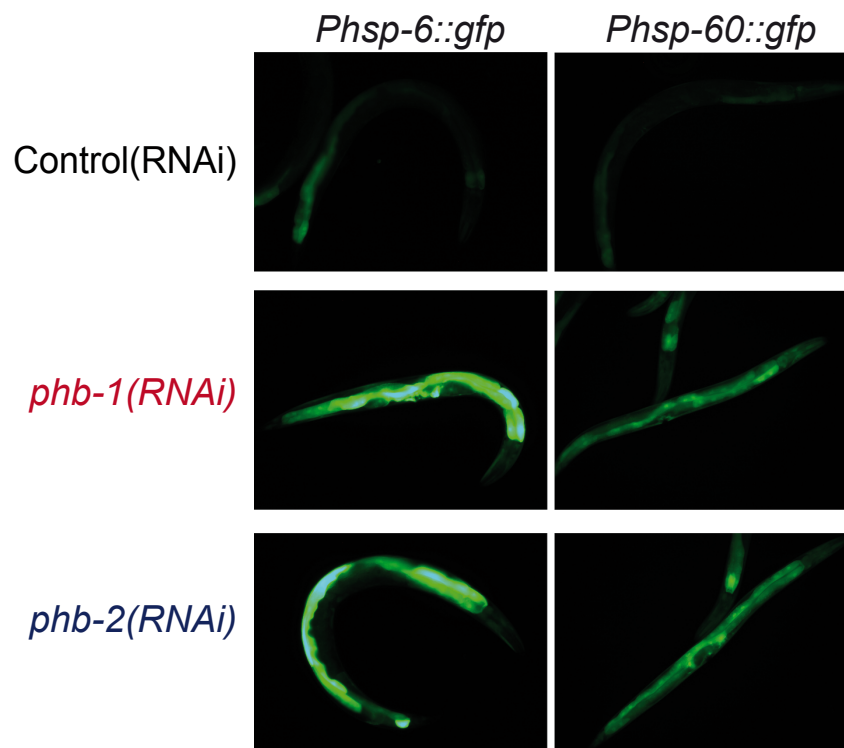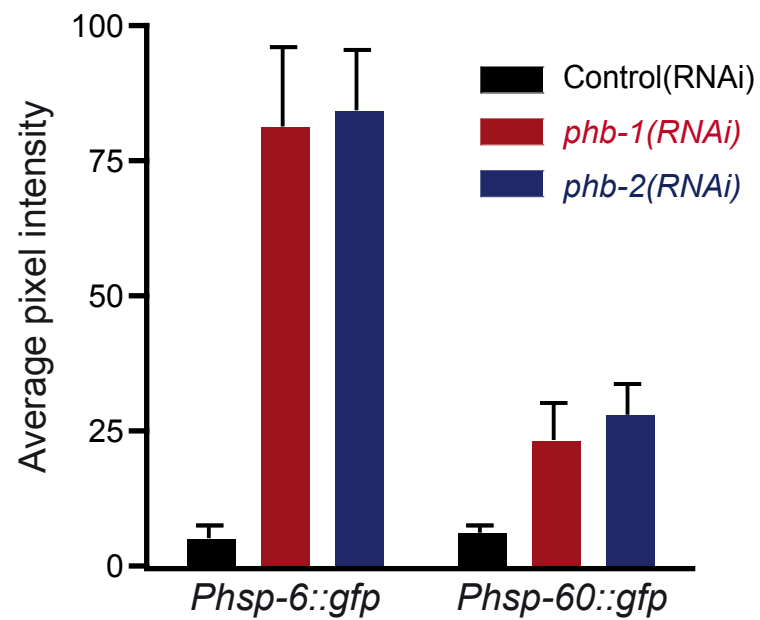

Supplement: Figure S3 — phb-1 and phb-2 RNAi induced the UPRmt. Left panel: Fluorescent microscopy of Phsp-6::gfp and Phsp-60::gfp animals subjected to RNAi with either phb-1 or phb-2. Worms were imaged at day 1 of adulthood. Depletion of either PHB-1 or PHB-2 induced similar levels of expression of the UPRmt reporters Phsp-6::gfp and Phsp-60::gfp. Right panel: Quantification of average pixel intensity of Phsp-6::gfp and Phsp-60::gfp animals subjected to RNAi with either phb-1 or phb-2 (n = 20 per strain and condition). (PDF) [file pone.0107671.s003.pdf]

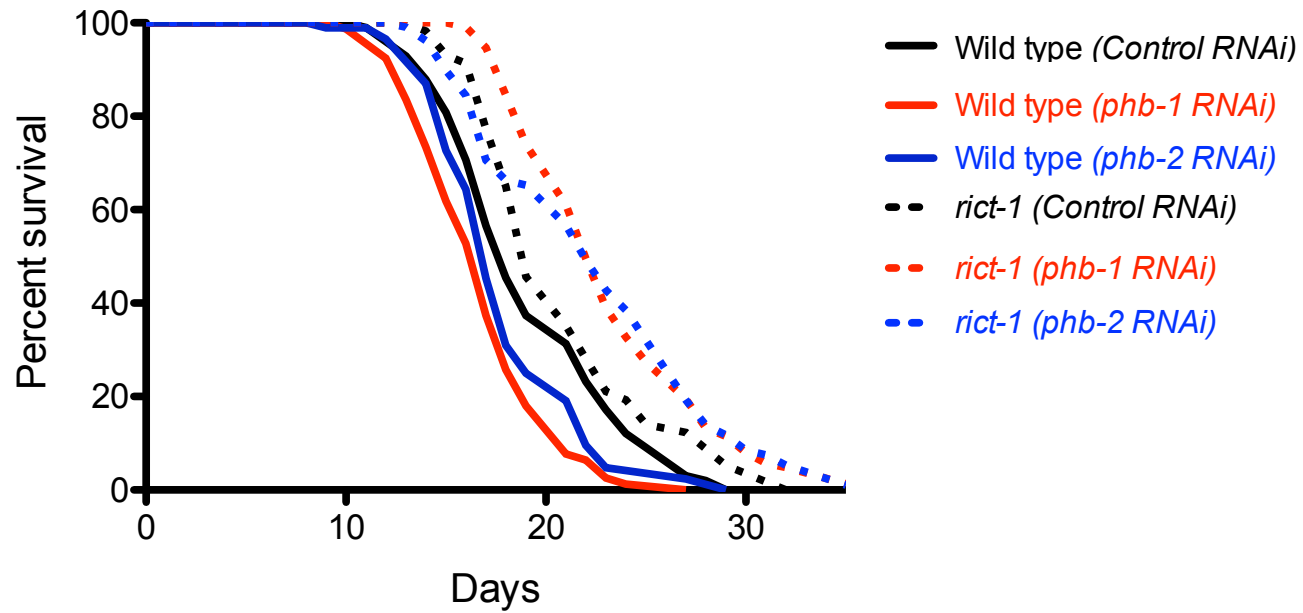

Supplement: Figure S4 — Prohibitin depletion extends the life span of rict-1 loss of function animals. Lifespan curves are represented as the percentage of animals remaining alive against animal age (days). Combined lifespan data from independent experiments are shown in Table S1. Prohibitin depletion by RNAi against phb-1 or phb-2, at 20°C extended the lifespan of rict-1(ft7) loss of function mutants. (PDF) [file pone.0107671.s004.pdf]

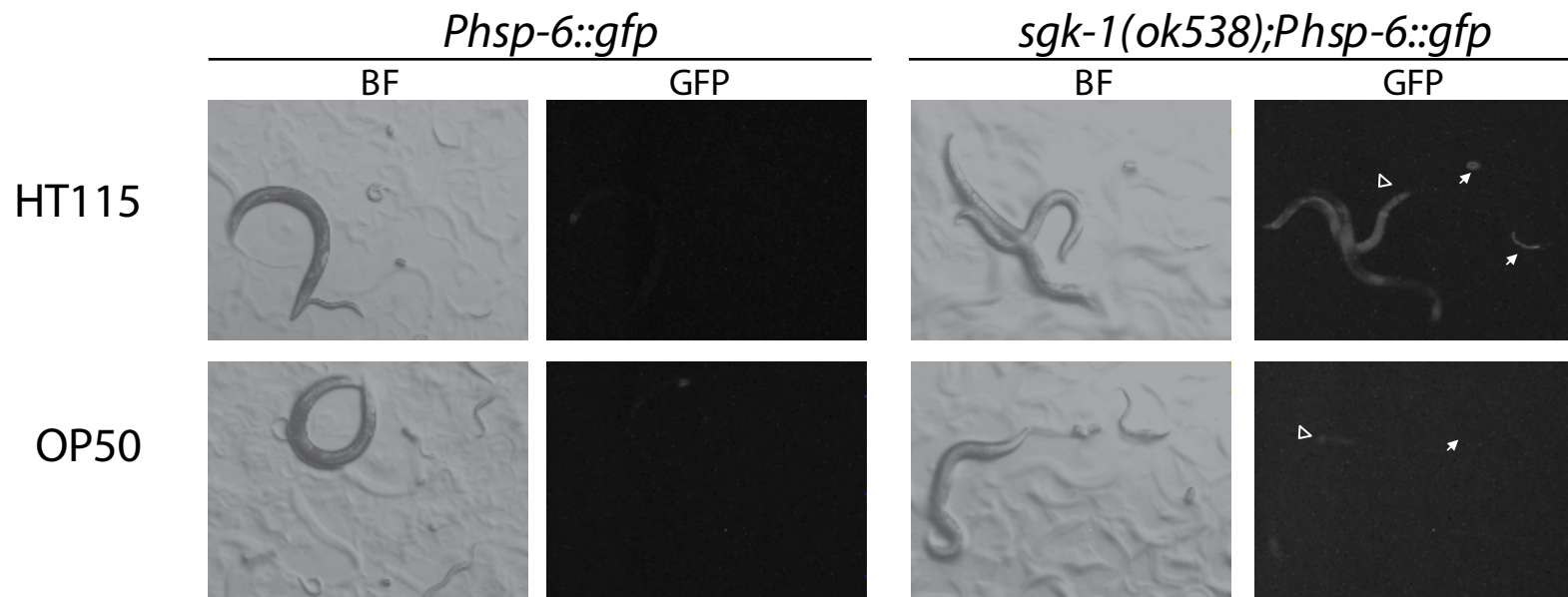

Supplement: Figure S5 — Induction of Phsp-6::gfp in sgk-1 mutants is more pronounced on HT115 in the F1 generation. Fluorescent microscopy of wild type; Phsp-6::gfp and sgk-1(ok538); Phsp-6::gfp animals grown on either HT115 or OP50 bacteria. Fluorescent stereoscope images of wild type; Phsp-6::gfp and sgk-1(ok538); Phsp-6::gfp (P0) and their progeny (F1). Bright field (BF) and fluorescent images are shown. Arrowheads point to P0 animals and arrows to F1 animals (egg and larvae). The induced expression of the Phsp-6::gfp reporter is evident in the P0 generation and becomes very strong in the F1 generation of sgk-1(ok538) animals grown on HT115 bacteria. (PDF) [file pone.0107671.s005.pdf]

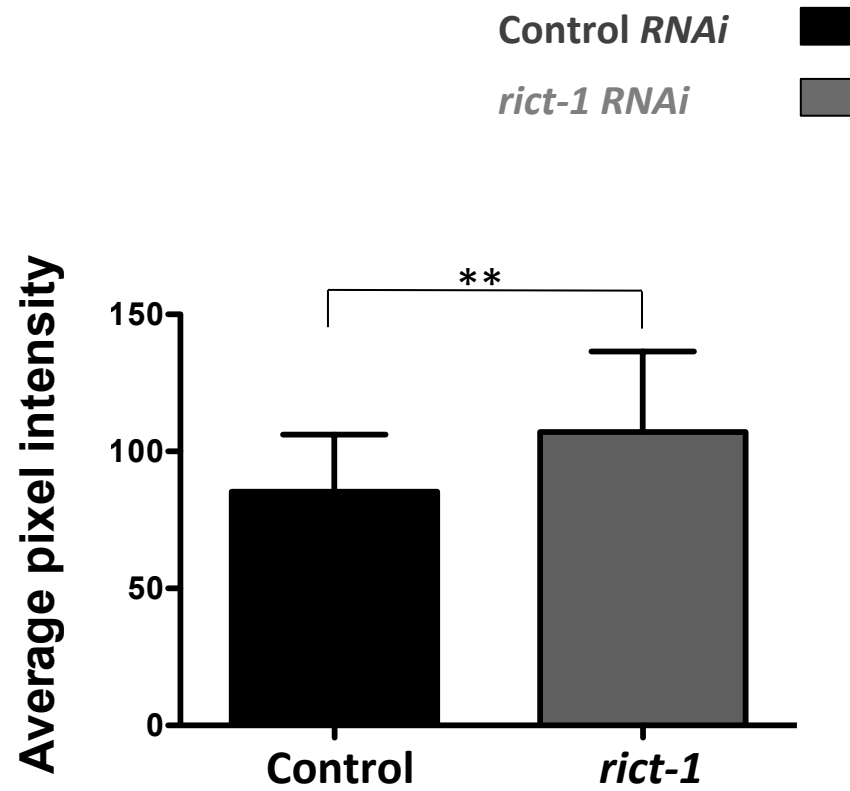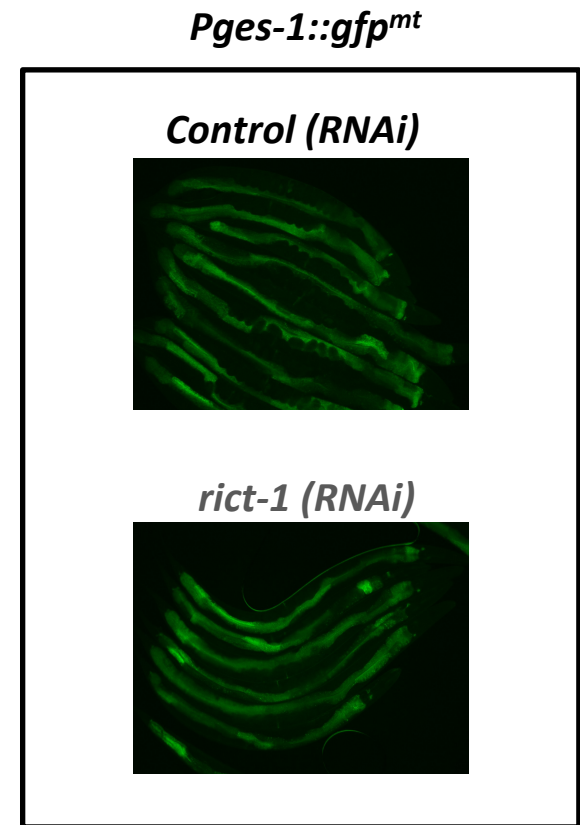

Supplement: Figure S6 — rict-1 RNAi increases the mitochondrial mass in the intestine. Fluorescent microscopy of Pges-1::gfpmt animals treated with empty vector pL4440 (control RNAi), or rict-1 RNAi (right panel) and graphical representation of the quantification of average pixel intensity under the corresponding conditions (left panel). Worms were imaged at the day 1 of adulthood. rict-1 depletion at 20°C increased intestinal mitochondrial mass as recorded by the intestinal mitochondrial reporter Pges-1::gfpmt. ** P value = 0.0057 (n = 22 for control RNAi, n = 28 for rict-1 RNAi). (PDF) [file pone.0107671.s006.pdf]

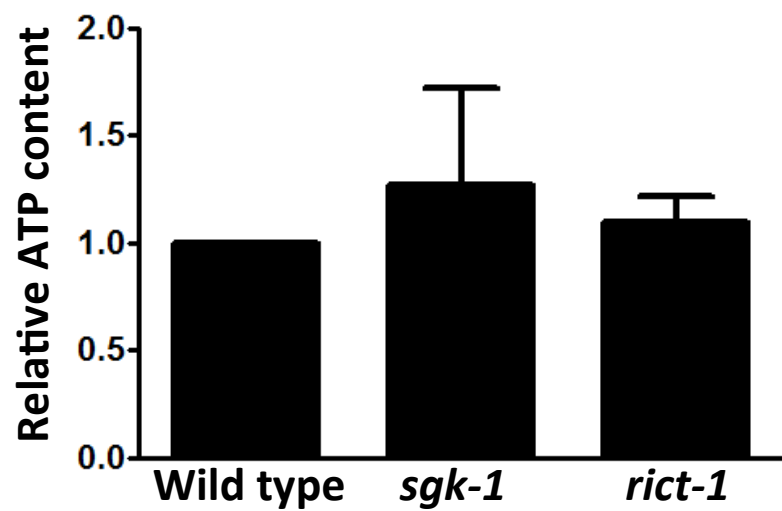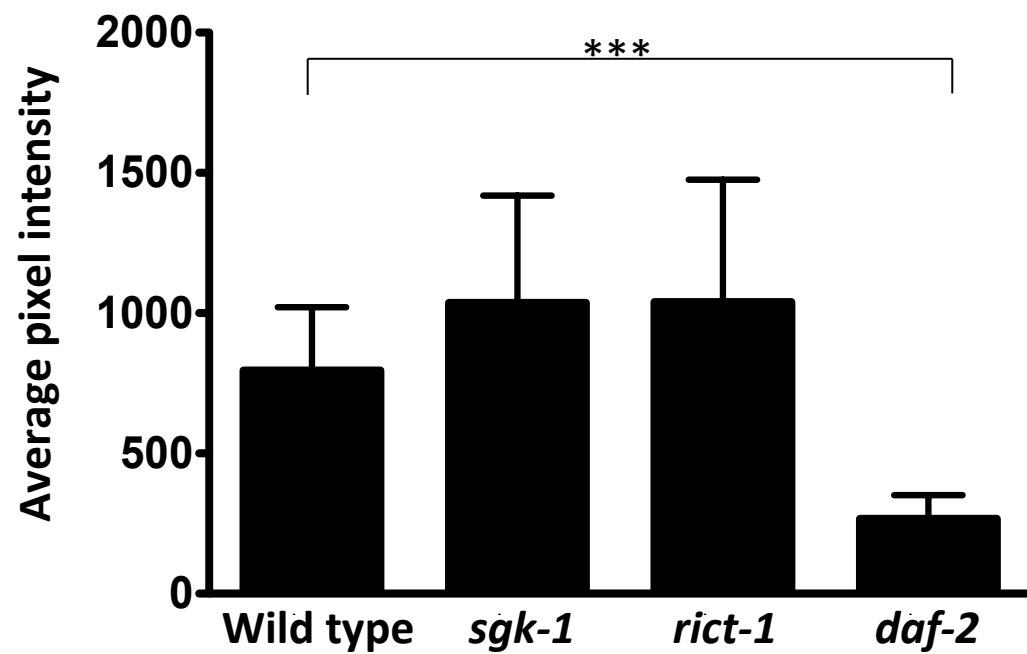

Supplement: Figure S7 — sgk-1 , rict-1 mutants do not effect ATP levels and the mitochondrial membrane potential. Left panel. Graphical representation of the ATP content (mM ATP/µg protein) normalized relative to the wild type control. Animals grown on HT115 bacteria containing the empty vector pL4440 at 20°C until day 10 of adulthood when they were collected for quantification of ATP levels and protein content. The graph represents data from three independent experiments. sgk-1(ok538) and rict-1(ft7) mutants do not have statistically significant different ATP content compared to the wild type control. Right panel. Graphical representation of the average pixel intensity of diS-C3 dye uptake measured by fluorescent microscopy in day 1 adult animals grown on HT115 bacteria containing the empty vector pL4440 at 20°C. Data from one representative experiment are shown. sgk-1(ok538) and rict-1(ft7) mutants did not cause a statistical alteration in the mitochondrial membrane potential while daf-2(e1370) mutants show a significant decrease. *** P value <0.0001. (PDF) [file pone.0107671.s007.pdf]

# MODEL

Mitochondrial stress observed by induction of the UPR<sup>mt</sup>

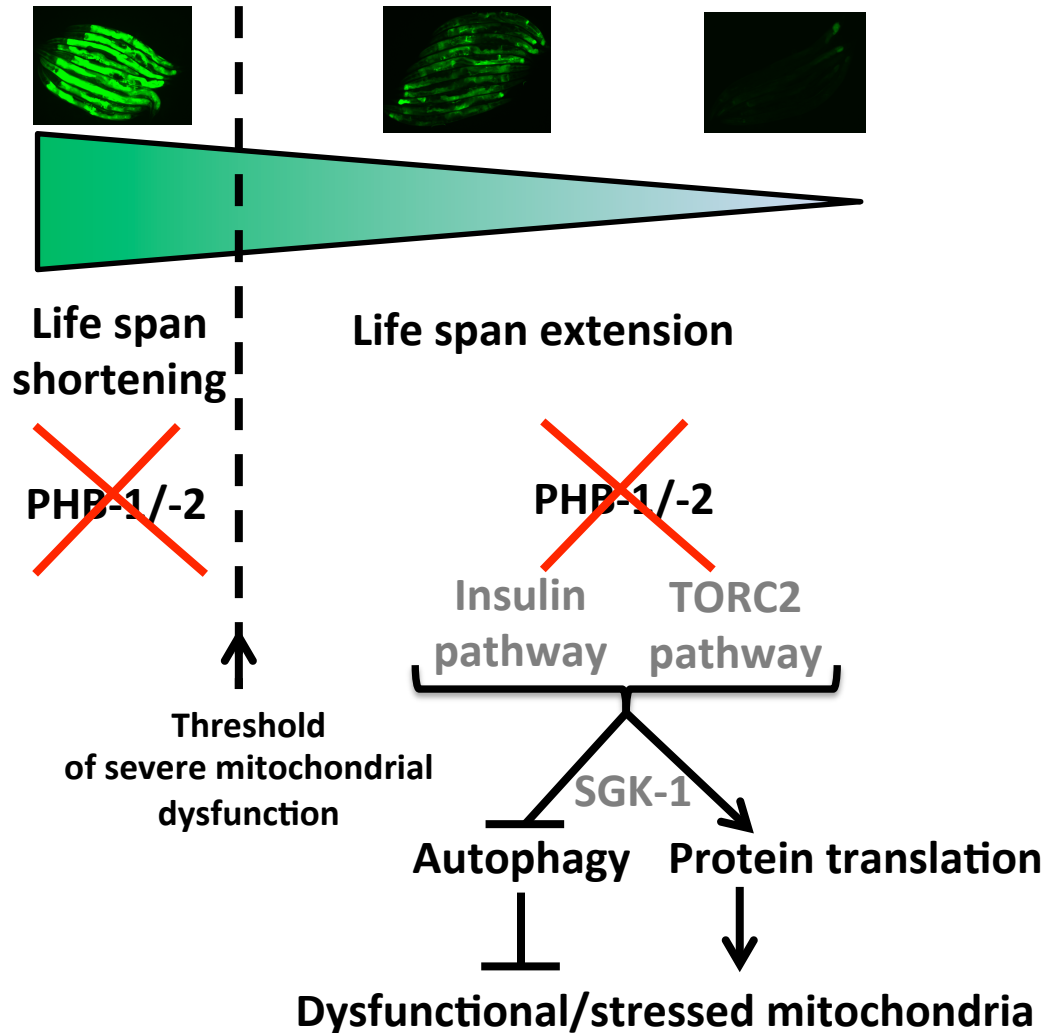

Supplement: Figure S8 — Proposed model for the differential role of prohibitins on life span. We propose that prohibitin depletion in a wild type background gives rise to severe mitochondrial dysfunction which over-induces mitochondrial stress response, resulting in early lethality for the organism. Conversely, in metabolically compromised background, like in daf-2, sgk-1 and rict-1 mutants, increased autophagy and/or reduction of protein synthesis is protecting the organism from excessive mitochondrial damage caused by the knockdown of prohibitins. This suppression of the mitochondrial damage/stress can be observed by suppression of the UPRmt. Under these conditions, the milder mitochondrial dysfunction upon prohibitin depletion could promote lifespan extension. (PDF) [file pone.0107671.s008.pdf]
