# Supplementary material for: Prohibitin-Mediated Lifespan and Mitochondrial Stress Implicate SGK-1, Insulin/IGF and mTORC2 in C. elegans
Source: PLoS One. 2014 Sep 29;9(9):e107671. doi: 10.1371/journal.pone.0107671 (PMC4180437; doi:10.1371/journal.pone.0107671)
Supplement: Table S1 — Summary of life span assays conducted for this study. Unless otherwise stated, all ageing experiments were performed on plates seeded with HT115(DE3) E. coli bacteria, carrying appropriate RNAi plasmid constructs (SD: standard deviation of the mean). ¶Maximum lifespan shown is the median lifespan of the longest-lived 10% of the animals assayed. †The number of confirmed death events, divided by the total number of animals included in lifespan assays is shown. Total equals the number of animals that died plus the number of animals that were censored (see Methods). The number of independent lifespan assays for each strain is shown in parentheses. *Compared to wild type animals subjected to control RNAi. ‡Compared to the corresponding mutant subjected to control RNAi. P values were calculated using the Log-rank (Mantel-Cox) Test. ∞Compared to wild type animals on HT115. n.s: not significant statistical difference. (PDF) [file pone.0107671.s009.pdf]

Supplementary Table 1

	Median ± SD	Max. ± SD¶	Deaths/Total†	P-value‡
N2	19.8±2.1	29.4±2.1	562/645(4)	
N2; *phb-1(RNAi)*	17.8±1.3	23.6±1.3	565/748(5)	< 0.0001
N2; *phb-2(RNAi)*	18.5±2.1	25.5±2.1	209/249(2)	< 0.0001
sgk-1(ok538)	25.8±4.3	33±4.9	519/798(4)	< 0.0001*
sgk-1(ok538); phb-1(RNAi)	29.5±4.4	42.7±6.5	510/705(4)	< 0.0001
sgk-1(ok538); phb-2(RNAi)	27±1.4	45.6±1.6	248/356(2)	< 0.0001
sgk-1(ft15)	19.7±4	28±4.7	425/552(3)	0.0055*
sgk-1(ft15); phb-1(RNAi)	16.3±4.0	25±7.0	380/542(3)	< 0.0001
sgk-1(ft15); phb-2(RNAi)	15.5±2.1	23.7±5.9	308/398(2)	< 0.0001
daf-2(e1370)	41.7±5.5	62.6±9.8	209/400(3)	< 0.0001*
daf-2(e1370); phb-1(RNAi)	60.3±10.0	86.7±12.9	379/423(3)	< 0.0001
daf-2(e1370); phb-2(RNAi)	52.5±6.4	72.7±27.1	219/272(2)	< 0.0001
daf-2(e1370); sgk-1(ok538)	68.3±2.9	95.8±14.4	208/460(3)	
daf-2(e1370); sgk-1(ok538); phb-1(RNAi)	90.7±8.7	112.8±0.8	228/467(3)	< 0.0001
daf-2(e1370); sgk-1(ok538); phb-2(RNAi)	89.5±12.0	120.3±6.2	186/298(2)	< 0.0001
rict-1(ft7)	21.5±3.54	27.8±3.54	170/447(2)	< 0.0001*
rict-1(ft7); phb-1(RNAi)	25.5±4.95	36.1±4.95	397/538(2)	< 0.0001
rict-1(ft7); phb-2(RNAi)	24.5±3.54	32.9±3.54	242/464(2)	< 0.0001
akt-1(ok525)	24.5±3.5	33.1±0.4	259/309(2)	< 0.0001*
akt-1(ok525); phb-1(RNAi)	22.5±3.5	31.5±5.5	160/245(2)	0.0062
akt-1(ok525); phb-2(RNAi)	23	31.9	87/100(1)	0.0289
akt-1(mg144)	20	24	45/53(1)	
akt-1(mg144); phb-1(RNAi)	15	23.5	115/127(1)	< 0.0001
akt-1(mg144); phb-2(RNAi)	20	24	69/93(1)	n.s
akt-2(ok393)	24±5.7	31±4.4	314/420(2)	0.0013*
akt-2(ok393); phb-1(RNAi)	21.5±6.4	34.3±2.3	361/544(2)	n.s
akt-2(ok393); phb-2(RNAi)	20	23.5	136/165(1)	n.s
age-1(hx546)	25±4	40±6	341/470(3)	
age-1(hx546); phb-1(RNAi)	24.3±2.5	41±0.8	267/506(3)	n.s
age-1(hx546); phb-2(RNAi)	23.7±0.6	39.3±5.5	382/544(3)	n.s
N2, HT115	17.0±0.0	24.5±0.7	278/319(2)	

N2, HT115 + FUdR	17.5±0.7	24.5±0.7	285/310(2)	n.s
sgk-1(ok538), HT115	24.5±0.7	28.0±0.0	112/313(2)	< 0.0001[∞]
sgk-1(ok538), HT115 + FUdR	18±1.4	25±1.4	244/273(2)	n.s [∞]

Supplementary Table 1. Summary of life span assays conducted for this study.

Unless otherwise stated, all ageing experiments were performed on plates seeded with HT115(DE3) *E. coli* bacteria, carrying appropriate RNAi plasmid constructs (SD: standard deviation of the mean).

¶Maximum lifespan shown is the median lifespan of the longest-lived 10% of the animals assayed.

†The number of confirmed death events, divided by the total number of animals included in lifespan assays is shown. Total equals the number of animals that died plus the number of animals that were censored (see Methods). The number of independent lifespan assays for each strain is shown in parentheses.

*Compared to wild type animals subjected to control RNAi.

‡Compared to the corresponding mutant subjected to control RNAi. P values were calculated using the Log-rank (Mantel-Cox) Test.

∞Compared to wild type animals on HT115.

n.s: not significant statistical difference.
